# CRISPR/Cas9-mediated mutagenesis of sweet basil candidate susceptibility gene *ObDMR6* enhances downy mildew resistance

**DOI:** 10.1371/journal.pone.0253245

**Published:** 2021-06-10

**Authors:** Jeremieh Abram R. Hasley, Natasha Navet, Miaoying Tian

**Affiliations:** Department of Plant and Environmental Protection Sciences, University of Hawaii at Manoa, Honolulu, HI, United States of America; Oklahoma Medical Research Foundation, UNITED STATES

## Abstract

Sweet basil (*Ocimum basilicum*) is an economically important allotetraploid (2n = 4x = 48) herb whose global production is threatened by downy mildew disease caused by the obligate biotrophic oomycete, *Peronospora belbahrii*. Generation of disease resistant cultivars by mutagenesis of susceptibility (S) genes via CRISPR/Cas9 is currently one of the most promising strategies to maintain favored traits while improving disease resistance. Previous studies have identified Arabidopsis *DMR6* (Downy Mildew Resistance 6) as an S gene required for pathogenesis of the downy mildew-causing oomycete pathogen *Hyaloperonospora arabidopsidis*. In this study, a sweet basil homolog of *DMR6*, designated *ObDMR6*, was identified in the popular sweet basil cultivar Genoveser and found to exist with a high copy number in the genome with polymorphisms among the variants. Two CRISPR/Cas9 constructs expressing one or two single guide RNAs (sgRNAs) targeting the conserved regions of *ObDMR6* variants were generated and used to transform Genoveser via *Agrobacterium*-mediated transformation. 56 T0 lines were generated, and mutations of *ObDMR6* were detected by analyzing the Sanger sequencing chromatograms of an *ObDMR6* fragment using the Interference of CRISPR Edits (ICE) software. Among 54 lines containing mutations in the targeted sites, 13 had an indel percentage greater than 96% suggesting a near-complete knockout (KO) of *ObDMR6*. Three representative transgene-free lines with near-complete KO of *ObDMR6* determined by ICE were identified in the T1 segregating populations derived from three independent T0 lines. The mutations were further confirmed using amplicon deep sequencing. Disease assays conducted on T2 seedlings of the above T1 lines showed a reduction in production of sporangia by 61–68% compared to the wild-type plants and 69–93% reduction in relative pathogen biomass determined by quantitative PCR (qPCR). This study not only has generated transgene-free sweet basil varieties with improved downy mildew resistance, but also contributed to our understanding of the molecular interactions of sweet basil-*P*. *belbahrii*.

## Introduction

Sweet basil (*Ocimum basilicum*) is an economically important crop that is cultivated for its favorable biochemical properties used in the culinary, pharmaceutical, cosmetic, and biodiesel industries [1–3]. *O*. *basilicum* is considered to be an allotetraploid (2n = 4x = 48), while different ploidy levels were identified in other species within the *Ocimum* genus [4]. Basil downy mildew (BDM) caused by the obligate biotrophic oomycete *Peronospora belbahrii* threatens the global production of sweet basil [5]. This devastating foliar disease can cause damage at nearly all growth stages of sweet basil [6]. BDM is characterized as a dark-hued mat observed on the abaxial surface of infected leaves caused by the emergence of sporangiophores and sporangia [5, 7]. On the adaxial surface, infected leaves develop chlorotic lesions that lead to necrosis and abscission of the leaves [7], resulting in unmarketable products.

Various measures have been explored for managing BDM, most of which having limited efficacy and/or practicality. Control of BDM has primarily relied on the frequent use of a limited number of fungicides [5], which has led to the evolution of fungicide-resistant strains [7, 8]. Deploying fans to reduce humidity during nocturnal periods [9], nocturnal illumination of basil plants [10], or solar heating during daytime [11], has been shown to significantly suppress and reduce the disease, but these measures are associated with increased labor and/or material cost. Considering the scale of commercial farming and the desirability for low-maintenance growing options of home gardeners, the most effective BDM control strategy is to utilize disease-resistant varieties. Resistance has been found in an exotic sweet basil variety Mrihani and other *Ocimum* species that differ vastly from popular sweet basil cultivars in ploidy, appearance, aroma and taste [12–14]. Transferring disease resistance to popular sweet basil varieties through traditional breeding is very time-consuming and faced with significant challenges, such as sexual incompatibility, hybrid F1 sterility, and linkage drag [7, 13, 15]. After eight years of breeding work, several BDM-resistant varieties were recently commercialized for sweet basil production [14]. However, the resistance of these varieties can be diminished under high disease pressure and comes from a single source, Mrihani [14], which suggests a potential risk of resistance breakdown if widely used. Additional efforts using technology that allows rapid breeding of disease resistant varieties with diverse resistance mechanisms are essential to sustain global sweet basil production.

Targeted mutagenesis of plant susceptibility (S) genes by clustered regularly interspaced short palindromic repeat (CRISPR)/ CRISPR-associated protein (Cas) mediated gene editing technology has been shown to be a promising way to breed disease-resistant varieties [16–19]. Plant genes that support pathogen fitness leading to successful infection and colonization are considered susceptibility (S) genes [20]. As many of the identified S genes are negative regulators of plant resistance or components required for a pathogen’s essential needs, mutating these S genes likely confers broad-spectrum and durable disease resistance [18, 21]. The CRISPR/Cas system allows for generation of complete gene knockout mutants as early as in the first generation of transgenic lines of a diploid or polypoid plant species [22–24], precise mutation, and generation of transgene-free mutant plants [23–25], which greatly speeds up the breeding and commercialization of a new variety.

The most widely used CRISPR system for plant genome editing is CRISPR/Cas9, which has been successfully used in genome editing of many plant species [16, 26]. This system functions through the complexing of the Cas9 endonuclease and a single guide RNA (sgRNA), which is a fusion of CRISPR RNA (crRNA) containing a programmable 20-nt RNA target sequence and a trans-activating crRNA (tracrRNA), at the DNA target site [27, 28]. The sgRNA-guided DNA cleavage activity of the Cas9 requires the presence of a protospacer adjacent motif (PAM, 5’-NGG-3’) immediately downstream of the 20-nt target sequence. Cas9 cleaves at 3-nt upstream of the PAM within the DNA target site, creating a double-strand break (DSB) [27]. Once the DSB is generated, the cell activates innate DNA repair mechanisms: non-homologous end joining (NHEJ), or homology-directed repair (HDR) when a homologous DNA template is present. The NHEJ DNA repair pathway is error prone and therefore exploited to generate mutations of short insertions or deletions (indels). We have recently demonstrated the successful application of CRISPR/Cas9 to sweet basil to generate transgene-free mutants using *Agrobacterium*-mediated transformation to deliver Cas9 and sgRNAs [24].

Arabidopsis *DMR6* (Downy Mildew Resistance 6) gene is a well characterized S gene against Arabidopsis downy mildew pathogen *Hyaloperonospora arabidopsidis*. *dmr6* mutants displayed complete resistance to *H*. *arabidopsidis* and enhanced resistance to *Fusarium graminearum* [29, 30]. *DMR6* encodes an oxidoreductase belonging to the 2-oxoglutarate 2(OG)-Fe(II) oxygenase superfamily [29], and was further characterized as a negative regulator of plant defense by hydrolyzing plant defense signaling molecule salicylic acid (SA) [31]. *DMR6* seems to be conserved in various plant species. Silencing of its potato ortholog *StDMR6* significantly enhanced resistance against the late blight-causing oomycete pathogen *Phytophthora infestans* [32]. The mutants of its tomato homolog *SlDMR6-1*, generated via CRISPR/Cas9, were more resistant to three tested plant pathogenic bacteria, including *Xanthomonas gardneri*, *X*. *perforans*, and *Pseudomonas syringae* pv. tomato, and the oomycete pathogen *Phytophthora capsici* [33]. Despite the potentially increased SA accumulation in these mutants, the tomato and potato mutants did not exhibit significant adverse effect on plant growth and development [32, 33]. As such, *DMR6* homologs represent ideal targets for mutagenesis to generate disease resistant varieties.

In the present study, we targeted the sweet basil homolog of *DMR6*, *ObDMR6*, using CRISPR/Cas9 to generate downy mildew-resistant sweet basil and meanwhile determine the role of *ObDMR6* in susceptibility to *P*. *belbahrii*. Two constructs expressing one or two sgRNAs were used to transform the popular sweet basil cultivar Genoveser via *Agrobacterium-*mediated transformation (AMT). We obtained multiple lines of transgene-free *ObDMR6* knockout mutants, which exhibited enhanced resistance to *P*. *belbahrii*.

## Materials and methods

### Plant materials and growth conditions

*O*. *basilicum* cultivar Genoveser (Enza Zaden) was used throughout the study. Juvenile plants were routinely grown in a temperature-controlled growth chamber set at 25°C with ambient humidity, and a 12 h photoperiod with light intensity at 60–100 μmol m^-2^ s^-1^. Similar conditions were applied to grow T0, T1, and T2 transgenic seedlings. T0 and T1 transgenic plants were grown to maturity in a greenhouse setting with normal conditions of 25–27°C and a 16 h photoperiod for seed production. In order to prevent cross-pollination, selfing bags were mounted onto flower stalks at the beginning of flowering.

### Identification of sweet basil *DMR6* homolog

The homolog of Arabidopsis *DMR6* (*AtDMR6*) in sweet basil (*Ocimum basilicum*), *ObDMR6*, was identified by TBLASTX search against the non-redundant transcriptomic sequence assembly generated from an *O*. *basilicum* cultivar, Dolly (Enza Zaden) (S1 Appendix), using the protein encoding sequence of *AtDMR6* (GenBank accession: NM_122361) as a query. Three significant hits were identified based on query coverage and E-value. To amplify *ObDMR6* from sweet basil cultivar Genoveser by PCR, we designed a pair of primers (*ObDMR6*-F: 5’-ATGGAAACGAAGGTCATTAGTG-3’; *ObDMR6*-R: 5’-CTAATTCTTGAATAGTTCCAGGCAG-3’) targeting the start and stop regions of the protein-encoding sequences of the identified transcripts from Dolly. Two PCR assays were preformed using Genoveser genomic DNA (gDNA) and complementary DNA (cDNA) as the templates and Phusion High-Fidelity DNA Polymerase (NEB) under the PCR conditions: initial denaturation at 98°C for 30 s; 35 cycles of 98°C for 30 s, 62°C for 15 s, and 72°C for 1.5 min; and a final extension at 72°C for 10 min. The resultant amplicons were separated by agarose gel electrophoresis, purified using QIAquick Gel Extraction (QIAGEN), cloned into pCR4Blunt-TOPO using Zero Blunt™ TOPO™ PCR Cloning Kit (ThermoFisher), and then subjected to Sanger sequencing. Sequence alignments were generated using CLUSTALX 2.1 [34].

### Selection of sgRNA target sequences for editing *ObDMR6* using CRISPR/Cas9

The 20-nt target sequences were identified by the Eukaryotic Pathogen CRISPR Guide RNA/DNA Design Tool (EuPaGDT) (http://grna.ctegd.uga.edu/) [35] using the protein-encoding sequence of a sequenced *ObDMR6* cDNA clone as a query and Dolly transcriptome as the custom genome for off-target analyses, with default parameters (sgRNA search parameter: 20 nt sequence with NGG immediately downstream of 3’ end; off-target search parameters: seed length including PAM as 15 nt, maximum number of mismatches as 3 nt). The potential off-targets were also checked by uploading the non-redundant transcriptomic assembly of two sweet basil varieties, Red Rubin and Tigullio [36], as a custom genome to the EuPaGDT. The candidate target sequences without a potential off-target, with both, a total and efficiency score, greater than 0.50, were subjected to secondary structure prediction using the web-based tool RNAStructure (http://rna.urmc.rochester.edu/RNAstructureWeb/Servers/Predict1/Predict1.html) [37]. The selected candidate target sequences predicted with less than three hydrogen bonds were further analyzed to identify the ones which were conserved in all identified *ObDMR6* variants in Genoveser, not spanning the introns, and closer to the start codon.

### Vector construction

The CRISPR/Cas9 constructs for gene editing of *ObDMR6* via *Agrobacterium*-mediated transformation were generated using pKSE401, a plant binary vector, as described by Xing et al. [38]. For generating the construct expressing only sgRNA1 (S1), an oligo pair (S1-Oligo-F: 5’-ATTGCACATACTGCAAAGAAGTT-3’; S1-Oligo-R: 5’-AAACAACTTCTTTGCAGTATGTG-3’) was used, where underlined letters indicate the target sequence of S1. The oligo pair was annealed in 1 × T4 DNA ligase buffer (NEB) in a 1.5 ml Eppendorf tube placed in a water bath, which started at 95°C and then slowly cooled down to room temperature, to produce a double-stranded DNA molecule with 4-nt 5’ overhangs on both strands. The annealed product was then ligated into the *Bsa*I-digested pKSE401. The resulting construct was labeled as pKSE401-S1.

For generating the construct expressing two sgRNAs (S1 and S2), four oligos (S1-DT1-BsF: 5’-ATATATGGTCTCGATTGCACATACTGCAAAGAAGTTGTT-3’; S1-DT1-F0: 5’-TGCACATACTGCAAAGAAGTTGTTTTAGAGCTAGAAATAGC-3’; S2-DT2-R0: 5’-AACGATCTGACTTTCGGATTACCAATCTCTTAGTCGACTCTAC-3’; S2-DT2-BsR: 5’-ATTATTGGTCTCTAAACGATCTGACTTTCGGATTACCAA-3’) were designed as described by Xing et al. [38], where underlined letters indicate sgRNA target sequences. A PCR assay was performed using the four oligos, Phusion High-Fidelity DNA Polymerase (NEB), and pCBC-DT1T2 [38] as a template under the PCR conditions: initial denaturation at 98°C for 2 min; 35 cycles of 98°C for 30 s, 71°C for 15 s, and 72°C for 1.5 min; and a final extension at 72°C for 7 min to generate an amplicon containing sgRNA1, U6-26 terminator, U6-29 promoter and sgRNA2 target sequence. The resultant amplicon was purified using QIAquick Gel Extraction (QIAGEN) and then cloned to pKSE401 via *Bsa*I. The resultant construct was labeled as pKSE401-S1S2.

### *Agrobacterium*-mediated transformation of sweet basil

The CRISPR/Cas9 *ObDMR6* gene-editing constructs, pKSE401-S1 and pKSE401-S1S2, were used separately to transform *Agrobacterium tumefaciens* strain EHA105 via electroporation. The resultant *Agrobacterium* strains were used for sweet basil transformation. The *Agrobacterium-*mediated transformation of explants derived from Genoveser plants was performed as described previously [24, 39].

### Detection of transgene integrations and *ObDMR6* mutations in transgenic plants

gDNA was isolated from regenerated sweet basil plants (T0 and T1) as described [24, 40]. Transgene integration and loss in T0 and T1 representative lines, respectively, were confirmed by a PCR assay using primer pair (U6-26p-F: 5’-TGTCCCAGGATTAGAATGATTAGGC-3’; S1-Oligo-R: 5’-AAACAACTTCTTTGCAGTATGTG-3’), Phusion High-Fidelity DNA Polymerase (NEB), and gDNA as a template under the PCR conditions: initial denaturation at 98°C for 30 s; 35 cycles of 98°C for 15 s, 61°C for 15 s, and 72°C for 1 min; and a final extension at 72°C for 10 min.

Identification of *ObDMR6* mutations in T0 and selected T1 transgene-free lines were performed by analyzing the Sanger sequencing chromatograms of a 495 bp PCR fragment spanning both S1 and S2 targets, using Interference of CRISPR Edits (ICE) v2 bioinformatic tool [41]. In order to detect mutations in all *ObDMR6* copies, a primer pair (*ObDMR6*-S1S2-F1: 5’-CGAGTTTCAACGTTAGAAAGGAGA-3’; *ObDMR6*-S1S2-R1: 5’-CCAGATGCTTTTGTCATCAACATTG-3’) exactly matching all identified *ObDMR6* variants (S2 Appendix) was used to amplify the 495 bp fragment. PCR assays were performed using the aforementioned primer pair, Phusion High-Fidelity DNA Polymerase (NEB), and gDNA as a template under the PCR conditions: initial denaturation at 98°C for 30 s; 35 cycles of 98°C for 15 s, 63°C for 15 s, and 72°C for 1 min; and a final extension at 72°C for 10 min. The PCR products were purified using QIAquick Gel Extraction (QIAGEN), followed by Sanger sequencing. The resultant chromatograms were analyzed using ICE v2 as described by Hsiau et al. [41].

For detailed analyses of the mutations in *ObDMR6* in selected T1 mutant lines, a 417 bp *ObDMR6* fragment spanning the S1/S2 target regions was subjected to amplicon deep sequencing. The *ObDMR6* fragment was amplified using primer pair (ObDMR6-S1S2_F2: 5’-CAACTGGAGAGACTATCTCAGGCT-3’; ObDMR6-S1S2_R2: 5’-TTCCCATCCTTGAGAACCTGAAG-3’), which was conserved in all identified *ObDMR6* variants and flanking S1/S2 target sites (S2 Appendix), under the following PCR conditions: initial denaturation at 98°C for 30 s; 35 cycles for 98°C for 15 s, 65°C for 15 s, and 72°C for 30 s; and a final extension at 72°C for 10 min. The amplicon was purified using QIAquick Gel Extraction (QIAGEN). Amplicon deep sequencing was performed using the pipeline for Next Generation Sequencing (NGS) Amplicon-EZ (GENEWIZ, Inc.) as described previously [24]. Over 300,000 high-quality reads were generated for each tested T1 transgenic line. Mutation types with ≥ 1% of total reads were analyzed in detail.

### *P*. *belbahrii* strain, infection conditions, and pathogen assay

The *P*. *belbahrii* strain was isolated from basil plants grown at Poamoho Research Station, Hawai’i. The propagation of *P*. *belbahrii* and inoculation onto sweet basil plants were performed following the protocol described previously [40], with minor modifications. Briefly, four 10 μl drops of sporangial suspension (1 x 10^4^ sporangia/ml) were inoculated on each of the first set of true leaves on 4-week-old seedlings. For quantification of pathogen biomass using quantitative PCR (qPCR), samples were harvested at 4 days post inoculation (dpi), stored, and processed following the protocol described by Shao and Tian [40]. For quantification of sporangia, samples were harvested at 9 dpi. Each sample consisted of 4 inoculated leaves from 3 plants to represent one biological replicate of three collected for each treatment. Leaf samples were weighed, submerged in 10 ml diH_2_O, and then vortexed for 1 min using Vortex-Genie2 (Thermo Fisher Scientific) at speed setting 8 to dislodge the sporangia. Sporangia of each sample were counted twice using a hemocytometer and then quantified as the number of sporangia per gram of leaf tissue. One-tailed *t*-test was performed using SAS software to determine the statistical significance of difference. Two representative leaves from each treatment were photographed at 9 dpi.

## Results

### Identification of *DMR6* homologs in *O*. *basilicum*

To identify the homolog(s) of Arabidopsis DMR6 (AtDMR6) (At5g24530) in sweet basil, we performed a TBLASTX search against the transcripts of a sweet basil cultivar Dolly (S1 Appendix). Three transcripts (comp38697_c0_seq1, comp38697_c1_seq3, and comp38697_c1_seq2) with significant homology to AtDMR6 were identified with E-values of 2e-150, 4e-124 and 2e-123, respectively. comp38697_c0_seq1 contained a full open reading frame (ORF) of 1011 bp, which was translated to a protein of 336 amino acids. Using the amino acid sequence of comp38697_c0_seq1, we performed BLASTP against Arabidopsis protein database Araport11 using The Arabidopsis Information Resource (TAIR) (https://www.arabidopsis.org) and the best hit was AtDMR6, suggesting that the transcript comp38697_c0_seq1 encodes a DMR6 ortholog in *O*. *basilicum*, thereby designated *ObDMR6*. comp38697_c1_seq3 and comp38697_c1_seq2 contained partial ORF sequences, which were highly similar to ORF comp38697_c0_seq1 with a few single nucleotide polymorphisms (SNPs) among them, suggesting that they are the variants of *ObDMR6* (S3 Appendix).

**Fig 1:**
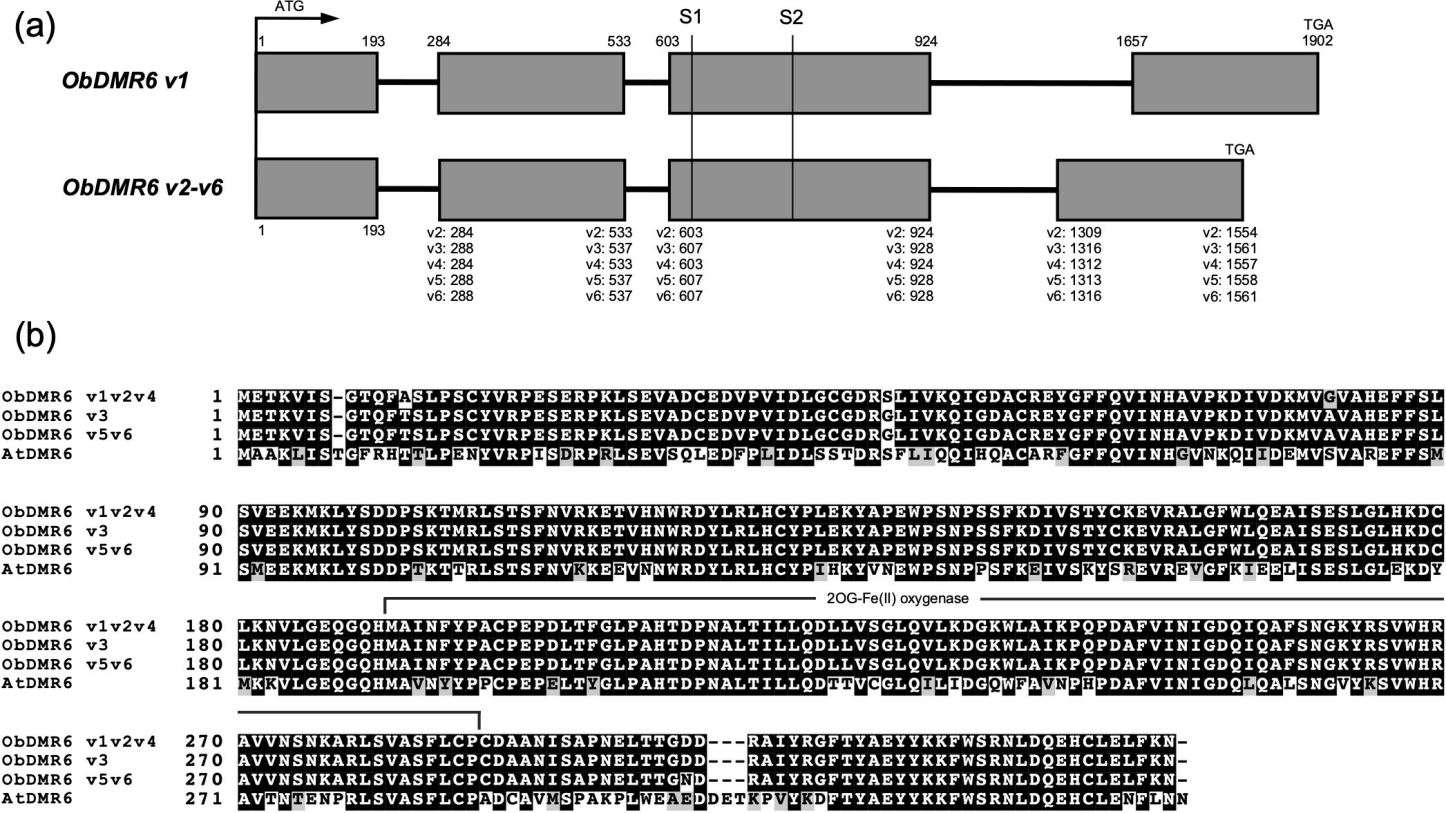
Schematic diagrams of gene structure of *ObDMR6* variants and their amino acid sequence alignment with AtDMR6. (a) Exons and introns of six *ObDMR*6 variants (v1-v6) starting from the start codon (ATG) to stop codon (TGA). Exons and introns are indicated using shaded boxes and solid lines, respectively. The numbers represent the nucleotide positions starting from the start codon. The positions of two sgRNA target sites (S1 and S2) are indicated. (b) Alignment of three distinct amino acid sequences of six ObDMR6 variants with AtDMR6. The conserved domain that defines 2OG-Fe(II) oxygenase superfamily of oxidoreductase (pfam03171) is indicated.

To perform gene editing in *O*. *basilicum* cultivar Genoveser, we amplified and sequenced the *ObDMR6* homologs in this cultivar, using the primers designed based on the ORF sequence of comp38697_c0_seq1. When using Genoveser gDNA as the template for PCR amplification, two distinct bands appeared after gel electrophoresis. DNA from each band was gel purified, and then cloned into pCR4Blunt-TOPO vector followed by Sanger sequencing. Four clones derived from the larger band contained identical sequences of 1902 bp in length. Nine clones derived from the smaller band resulted in five unique sequences ranging from 1554–1561 bp with various SNPs among them (S2 Appendix). The six unique *ObDMR6* gDNA sequences were designated as *ObDMR6 v1* to *v6*, which were deposited to NCBI GenBank with accession numbers MW776600-MW776605. To identify the introns/exons of these *ObDMR6* gene variants, we also amplified the cDNA of *ObDMR6* and cloned into pCR4Blunt-TOPO. Two clones were sequenced and contained identical sequences of 1011 bp (GenBank accession number: MW776606). Exons/introns of *ObDMR6* variants were predicted by aligning the sequenced *ObDMR6* cDNA sequence with the gDNA sequences using CLUSTALX 2.1. From the start codon to stop codon, four exons and three introns were identified in all *ObDMR6* gDNA sequences (Fig 1A, S2 Appendix). The *ObDMR6* gDNA sequences from the larger variant *(ObDMR6 v1)* and the smaller variants *(ObDMR6 v2-6)* differ primarily within the third intron (Fig 1A). Five unique ORF sequences representing three unique amino acid sequences were identified from these variants, with *ObDMR6 v*3 and the sequenced cDNA, *v*5 and *v*6, and the remaining three, *v*1, *v*2 and *v*4, encoding identical proteins, respectively. All three protein sequences shared high homology with AtDMR6 (Fig 1B). BLASTP against Arabidopsis protein database Araport11 returned AtDMR6 as the best hit. NCBI Conserved Domain search identified a functional domain (located from amino acid 191 to 287 of ObDMR6 variants) that defines 2OG-Fe(II) oxygenase superfamily of oxidoreductase (pfam03171) (Fig 1B). In total, we identified and sequenced *ObDMR6* homolog(s) in *O*. *basilicum* cultivar Genoveser. The six variants were identified from a limited number of clones suggesting that a greater number of *ObDMR6* copies likely exist in the genome.

### CRISPR/Cas9 design and generation of gene-editing constructs

**Fig 2:**
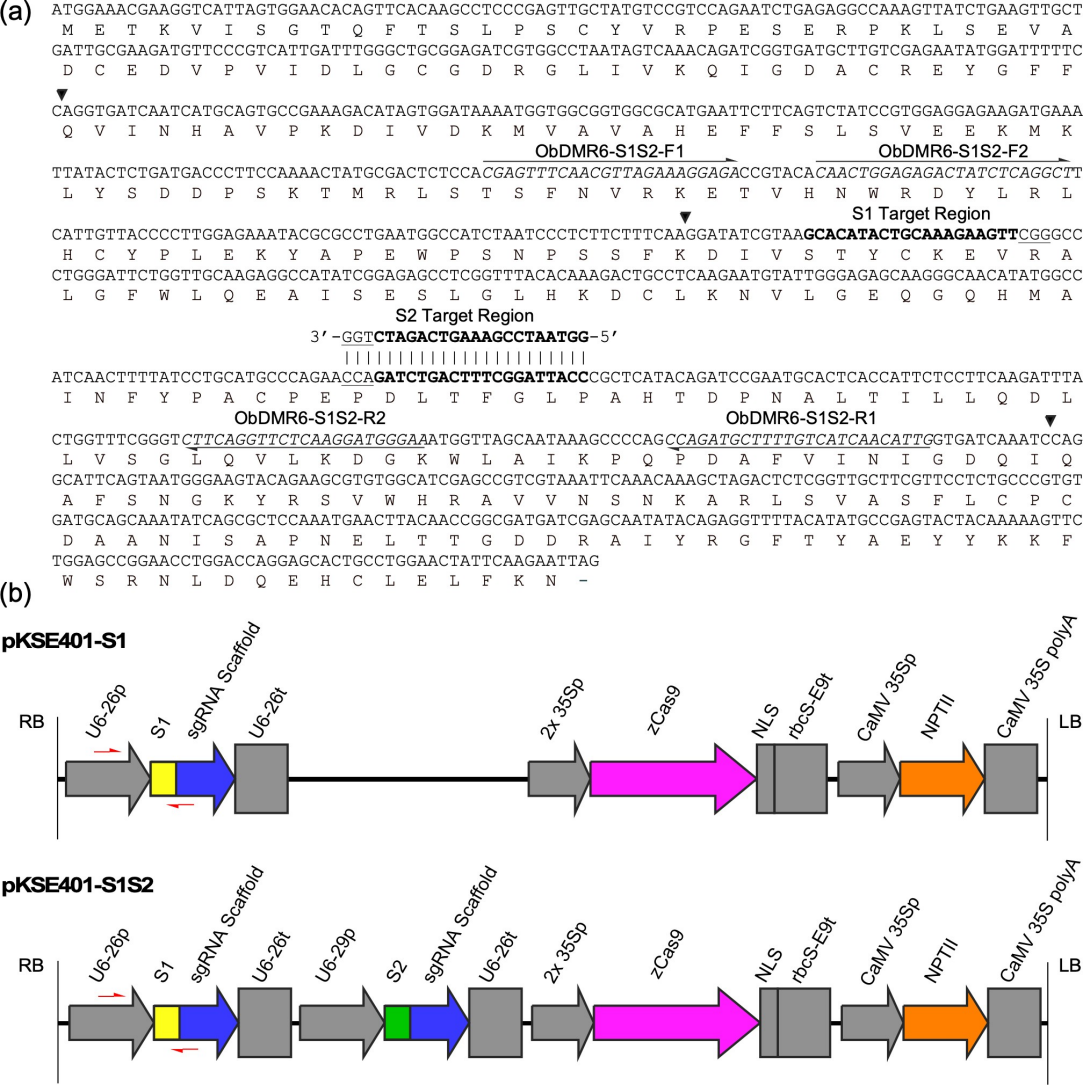
sgRNA target sites and constructs used for targeted mutagenesis of *ObDMR6*. (a) The protein encoding sequence and the translated amino acid sequence derived from a cDNA clone of Genoveser *ObDMR6* with two 20-nt sgRNA target sequences (S1 and S2) marked in bold and PAM underlined. The locations of three introns are indicated with black arrow heads. The primers (ObDMR6-S1S2-F1/R1, ObDMR6-S1S2-F2/R2) were used for the amplification of *ObDMR6* fragments for mutation analyses are indicated. (b) Schematic representations of expression cassettes within the T-DNA of pKSE401-S1 and pKSE401-S1S2, which were designed to express sgRNA1 (S1) only and two sgRNAs (S1 and S2), respectively. The elements were described in Xing et al. [38]. The primer pairs (U6-26p-F and S1-Oligo-R) used for detecting the transgene integration in plants are indicated by red arrows.

To be able to potentially mutate all copies of *ObDMR6* in Genoveser, we selected two 20-nt sgRNA target sequences that are conserved in all identified *ObDMR6* sequences, located at the third exon (Figs 1A and 2A, S2 Appendix), with a total and efficiency score greater than 0.50, and predicted to have a secondary structure of low complexity (S4 Appendix). sgRNA1 target (S1) (5’-GCACATACTGCAAAGAAGTT-3’) is on the sense strand, and sgRNA2 target (S2) (5’-GGTAATCCGAAAGTCAGATC-3’) is 132 bp downstream of S1 target on the complementary strand (Fig 2A). The GC content was 40% and 45% for S1 and S2, respectively. No potential off-targets were identified in the transcriptome of Dolly and the non-redundant transcriptomic assembly of two sweet basil varieties, Red Rubin and Tigullio [36]. To perform gene editing using *Agrobacterium*-mediated transformation, two plasmids were constructed where pKSE401-S1 was designed to express the sgRNA1, and pKSE401-S1S2 was designed to express sgRNA1 and sgRNA2 simultaneously. Arabidopsis U6-26 promoter and U6-29 promoter were used to drive the expression of sgRNA1 and sgRNA2, respectively (Fig 2B). Both constructs also contained the cassettes to express maize-codon optimized Cas9 under the control of the double CaMV 35S promoter, and NPTII gene under the control of CaMV 35S promoter for selection of transgenic plants (Fig 2B).

### Targeted mutagenesis of *ObDMR6* in T0 transgenic plants

**Fig 3:**
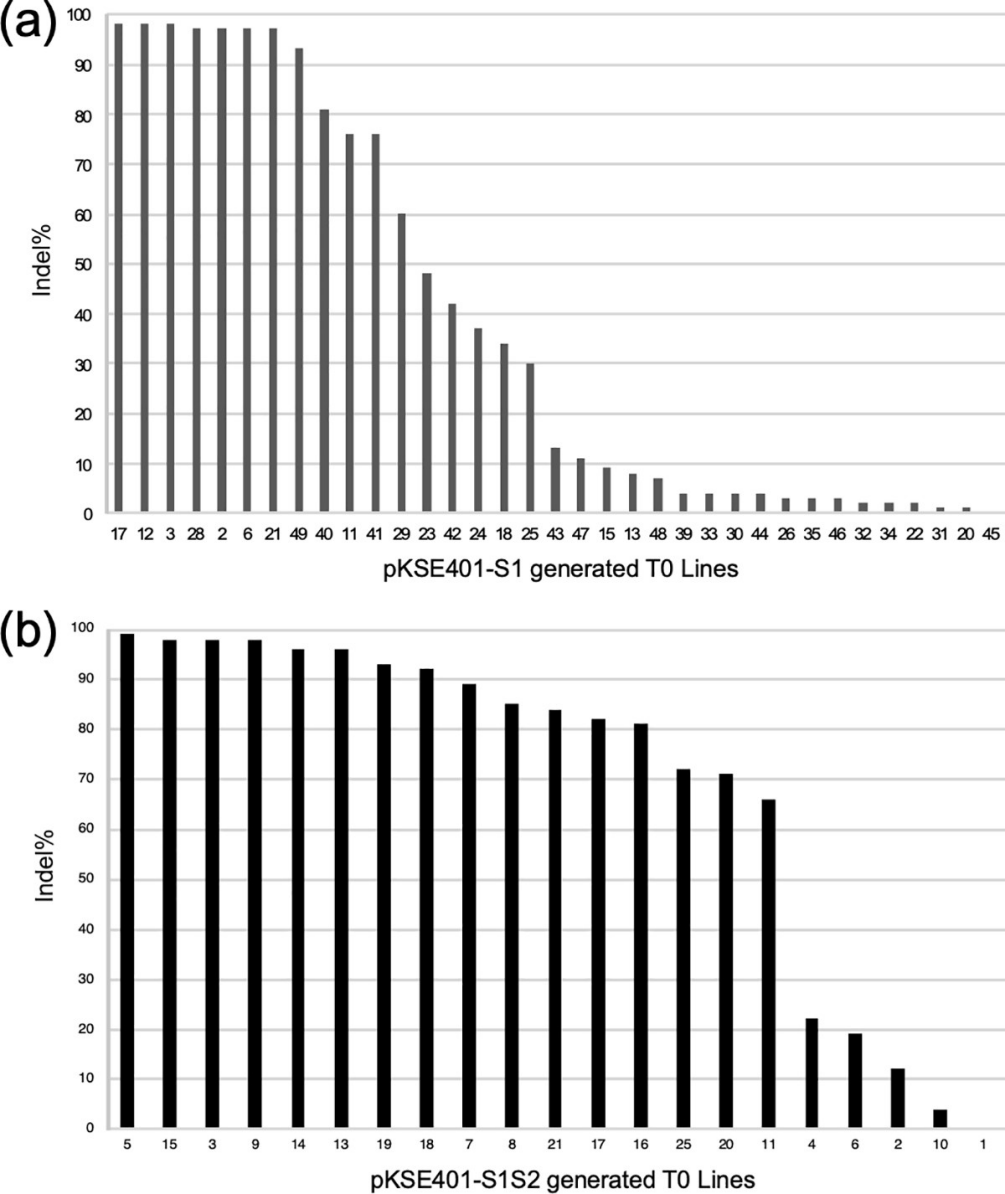
Indel percentages of T0 lines determined using interference of CRISPR edits v2 bioinformatic tool. (a) 35 lines transformed with pKSE401-S1. (b) 21 lines transformed using pKSE401-S1S2. Indel percentages of lines transformed with pKSE401-S1S2 consist of the mutations at both target sites.

To generate *Obdmr6* mutants with the goal to enhance basil downy mildew disease resistance, *A*. *tumefaciens* EHA105 strains carrying pKSE401-S1 and pKSE401-S1S2, respectively, were used to transform *O*. *basilicum* cultivar Genoveser via *Agrobacterium*-mediated transformation with leaf discs excised from the first pair of true leaves of 3-week-old seedlings as explants. 35 putative transgenic lines (T0) using pKSE401-S1 and 21 T0 lines using pKSE401-S1S2 were regenerated and grew to maturity, in addition to a number of lines that were lost during the acclimatization period. All 56 T0 lines were subjected to mutation analyses. A 495 bp *ObDMR6* fragment spanning both target sites was amplified from these lines using the primers *ObDMR6*-S1S2-F1/R1 (Fig 2A, S2 Appendix), followed by Sanger sequencing. Insertions/deletions (indels) were detected by analyzing the sequencing chromatograms using ICE v2 [41]. Varying levels of mutations in *ObDMR6* were identified in 34 of 35 (97%) T0 lines transformed with pKSE401-S1 and 20 of 21 (95%) T0 lines transformed with pKSE401-S1S2 (Fig 3). Seven pKSE401-S1 transgenic lines (S1: 2, 3, 6, 12, 17, 21, 28) and six pKSE401-S1S2 transgenic lines (S1S2: 3, 5, 9, 13, 14, 15) had an indel percentage greater than 96%, suggesting a near-complete knockout (KO) of *ObDMR6* in the T0 generation (Fig 3, S5 Appendix). 28 of 56 (50%) T0 lines had an indel percentage over 50%. For pKSE401-S1S2 lines, we further determined the indel percentage at each target site. Although mutations at the S1 target site were detected in 20 out of 21 T0 lines carrying both sgRNAs, only four of these lines (S1S2: 3, 9, 13, 14) contained mutations in the S2 target site (S5 Appendix). These four lines contained mutations at both target sites, with indel percentages (83%, 84%, 74% and 84% respectively) at S1 much higher than the indel percentages (17%, 14%, 22% and 32% respectively) at S2 (S5 Appendix).

Based on indel types and percentage, we selected two S1 (S1:3, 49) and one S1S2 (S1S2:15) T0 lines for further analyses into later generations. They had indel percentages of over 90% and distinct mutation profiles (indel types and the corresponding percentages) (S5 Appendix). S1:3 contained 98% indels, with a 1 bp insertion and two types of 3 bp deletion. This line appeared to contain a similar mutation profile as six other S1 lines (S1: 2, 6, 12, 17, 21, 28) with high percentage of indels. S1:49 contained 93% indels, with a 1 bp insertion and an 8 bp deletion. S1S2:15 had 98% indels, all of which occurred at S1 target site, with 3 bp and 4 bp deletions as the predominant mutation types. These lines were self-fertilized and grown to the T1 generation.

### Selection of transgene-free *Obdmr6* mutants in T1 generation

**Fig 4:**
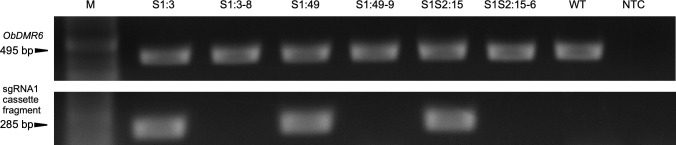
Characterization of transgene-free T1 plants expressing sgRNA1 only (S1) and two sgRNAs (S1S2). Agarose gel images showing PCR amplification of a 495 bp *ObDMR6* fragment (upper panel) and part of sgRNA1 cassette (lower panel) using primers shown in Fig 2. T1 plants S1:3–8, S1:49–9 and S1S2:15–6 were derived from T0 lines S1:3, S1:49 and S1S2:15, respectively. WT, wild-type plant; NTC, no template control; M, NEB 100 bp ladder.

To identify T1 *Obdmr6* mutants that have lost the transgene through genetic segregation, we preformed PCR assays using primers targeting U6-26p and sgRNA1 target sequence (Fig 2B) on T1 plants derived from T0 lines (S1:3, S1:49 and S1S2:15). For each line, we analyzed 9–20 T1 plants, and identified 3–4 transgene-free T1 plants. We selected one representative T1 plant from each T0 line, including S1:3–8, S1:49–9 and S1S2:15–6, to confirm the loss of the transgene. As shown in Fig 4, we were able to amplify the 495 bp *ObDMR6* fragment using primers *ObDMR6*-S1S2-F1/R1 from all tested T0, T1 and wild-type (WT) plants, suggesting the integrity of template DNA. While the 285 bp transgene fragment was detected in all tested T0 and WT plants, we failed to detect it in the tested T1 plants in repeated assays, suggesting that these T1 plants are indeed transgene-free.

**Fig 5:**
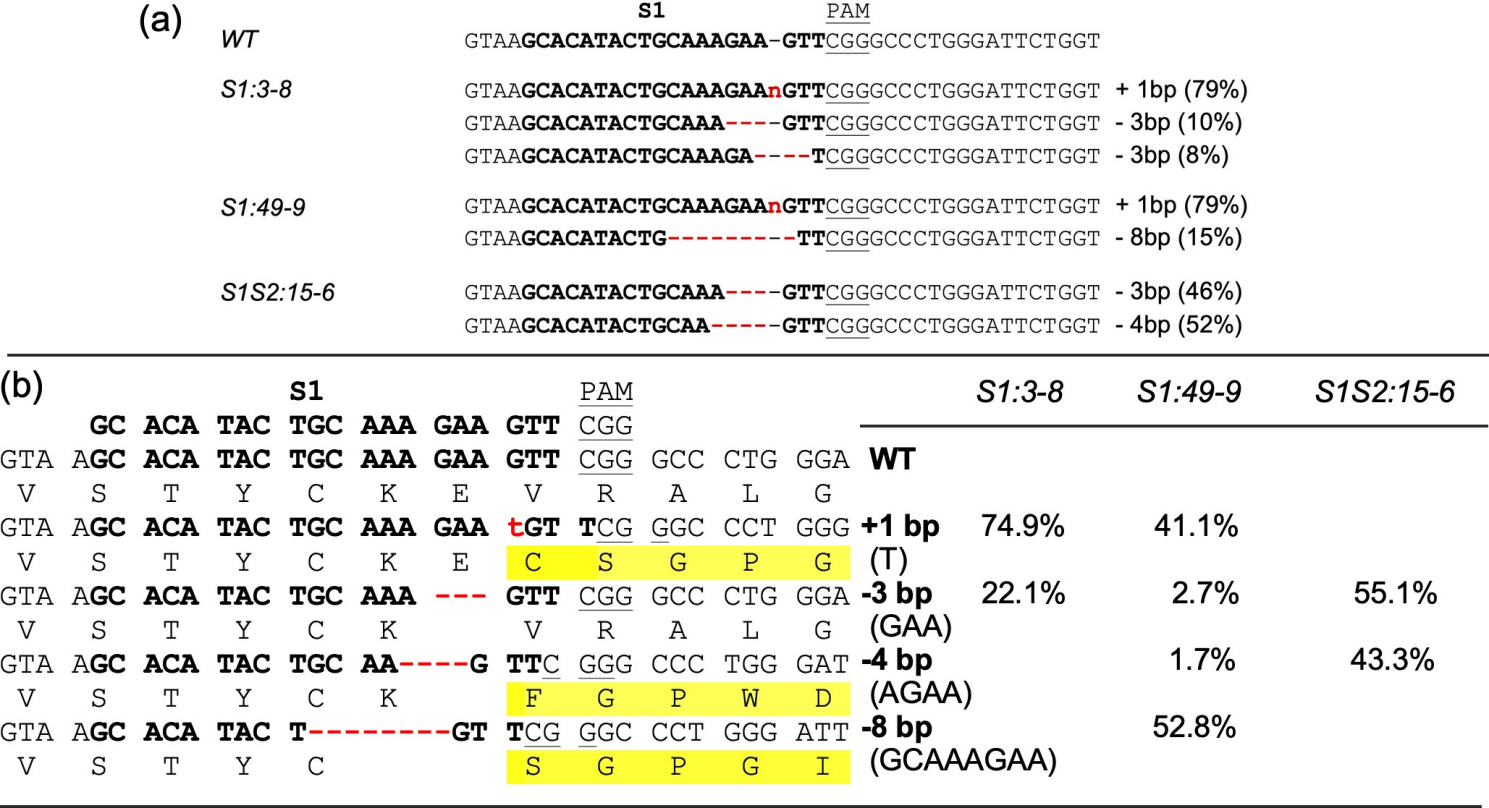
Mutations in transgene-free T1 plants at the sgRNA1 (S1) target site and the resulted amino acid sequence changes. (a) Indel types and the corresponding percentages in indicated T1 plants, detected using ICE v2 bioinformatic tool. (b) Mutations, the resultant amino acid sequence changes, and the corresponding percentage of reads, determined by amplicon deep sequencing. WT, the shown sequence fragment derived from the wild-type plant. The 20-nt S1 target sequence is shown in bold and the PAM underlined. The insertions are shown in red letter in lower case. “n” represents an inserted nucleotide that ICE v2 algorithm did not specify. The deletions are shown in red dashed lines.

The above selected transgene-free T1 plants were subjected to mutation analyses first using ICE analyses of Sanger sequencing chromatograms of the 495 bp *ObDMR6* fragment amplified using primers ObDMR6-S1S2_F1/R1 (Fig 2A, S2 Appendix). They all contained the major mutations initially detected in their corresponding T0 plants, and no wild-type *ObDMR6* sequence was detected (Fig 5A, S5 Appendix). These plants were further subjected to amplicon deep sequencing of a 417 bp *ObDMR6* fragment amplified using primers ObDMR6-S1S2_F2/R2 (Fig 2A, S2 Appendix). Over 300,000 high-quality reads were generated per line and mutation types with ≥ 1% of total reads were analyzed. Four major types of mutations, 1 bp “T” insertion and deletions of GAA (-3 bp), AGAA (-4 bp), GCAAAGAA (-8 bp), were detected at the S1 target site. GAA (-3 bp) deletion led to the loss of one amino acid glutamic acid (E). All other types of mutations led to frameshift and generation of a premature stop codon, resulting in shorter and drastically altered amino acid sequences (Fig 5B). S1:3–8 contained the “T” insertion (74.9% of total reads) and deletion of GAA (-3 bp) (22.1%). S1:49–9 contained 1 bp “T” insertion (41.1%), deletion of GCAAAGAA (-8 bp) (52.8%), and small percentage of deletions of GAA (-3 bp) (2.7%) and AGAA (-4 bp) (1.7%). S1S2:15–6 contained deletions of GAA (-3 bp) (55.1%) and AGAA (-4 bp) (43.3%). No significant number of reads corresponding to WT (≤0.5%) was identified in the amplicon sequencing data. Overall, similar results were obtained from ICE analyses of Sanger sequencing chromatograms and amplicon deep sequencing, both suggesting that T1 plants S1:3–8, S1:49–9 and S1S2:15–6 are transgene-free *Obdmr6* knockout mutants. These T1 lines were grown to produce T2 seeds.

### *Obdmr6* mutants exhibit enhanced resistance to *P*. *belbahrii*

**Fig 6:**
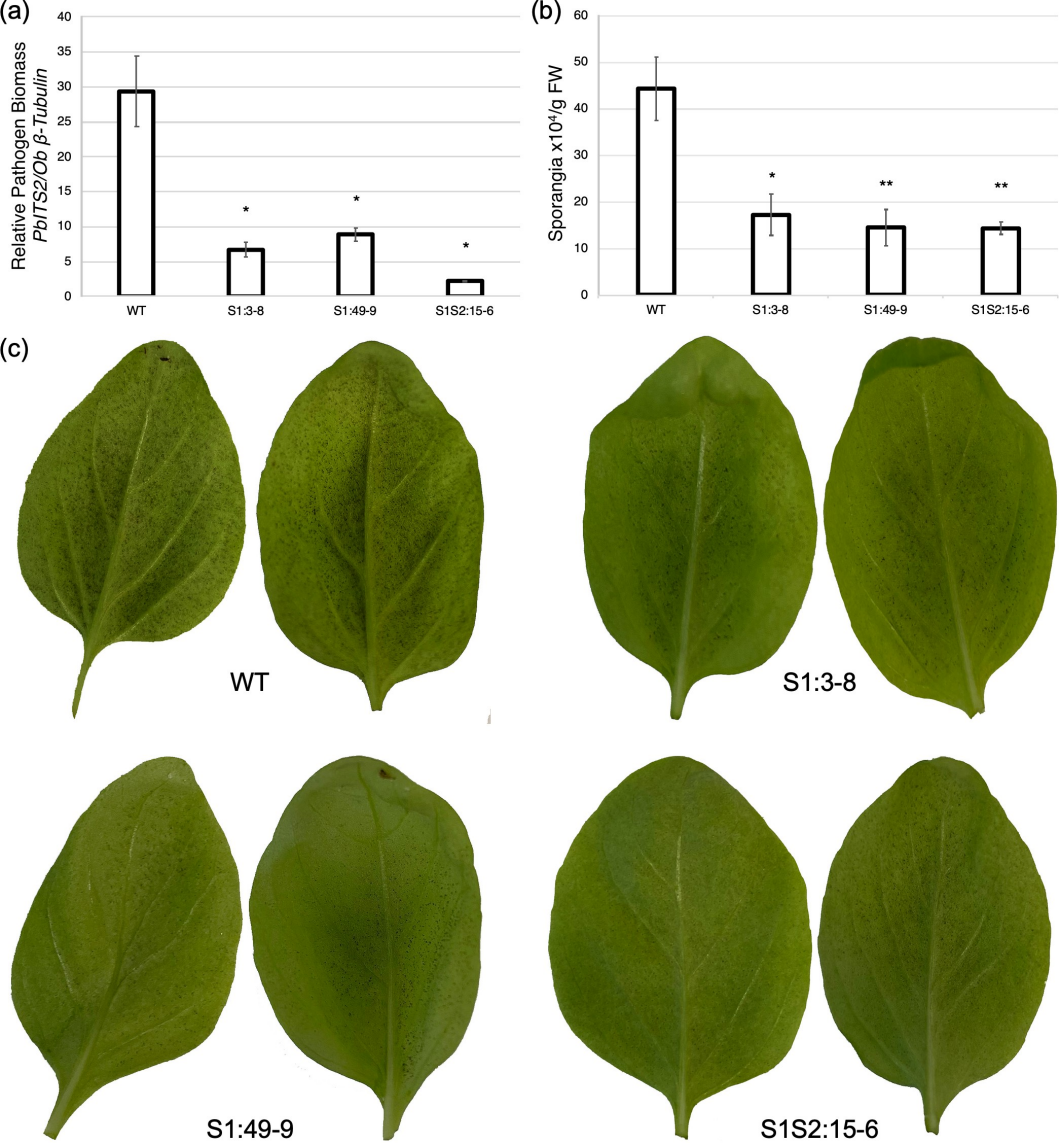
Mutation of *ObDMR6* in T2 complete knockout mutants enhances resistance to *Peronospora belbahrii*. (a) Pathogen biomass determined by qPCR. Infected leaf samples were collected from the wild-type (WT) and T2 plants derived from indicated T1 lines at 4 days post inoculation (dpi). The pathogen biomass was quantified as the ratio of amplification of *P*. *belbahrii* ITS2 relative to *O*. *basilicum* β-tubulin. Values represent mean ± standard error of three biological replicates with each consisting of two technical replicates. (b) Production of sporangia on inoculated leaves at 9 dpi, calculated as the number of sporangia per gram of fresh weight. Values represent mean ± standard error of three biological replicates. Statistically significant differences between the mutants and WT were determined by one-tailed t test, with one asterisk (*) indicating P < 0.05 and two asterisks (**) indicating P < 0.01. (c) Differential basil downy mildew disease levels of WT and T2 plants derived from indicated T1 lines shown with sporulation on the abaxial side of the leaves at 9 dpi. The experiments were repeated three times with similar results.

To determine whether the mutation of *ObDMR6* alters resistance against basil downy mildew, *P*. *belbahrii* was inoculated onto the first set of true leaves of 4-week-old T2 plants derived from the above identified transgene-free *ObDMR6* knockout T1 lines. We quantified the pathogen biomass at 4 dpi using qPCR and production of sporangia at 9 dpi. For quantification of pathogen biomass, the relative pathogen biomass was determined as the amplification of *P*. *belbahrii internal transcribed spacer 2* (*PbITS2*) relative to the amplification of *O*. *basilicum β-tubulin* [40]. The pathogen growth on *Obdmr6* mutant lines S1:3–8, S1:49–9 and S1S2:15–6 decreased by 69–93% when compared to the WT plants (Fig 6A). For quantifying production of sporangia, we counted the sporangia dislodged from infected leaves and determined the number of sporangia per gram of leaf tissues. The sporangia produced on *Obdmr6* T2 mutants were 61–68% lower than WT (Fig 6B), which was consistent with the visual symptoms shown on the underside of the leaves (Fig 6C). These results indicated that mutation of *ObDMR6* significantly enhanced basil downy mildew resistance. No significant difference in plant growth and development was observed between *Obdmr6* mutants and WT.

## Discussion

Many pathogens exploit host factors to suppress plant defense response, and facilitate nutrient uptake and accommodation in the hosts [20]. It is well accepted that disrupting these S genes represents an effective way to generate disease resistance [18, 42]. Sweet basil S genes that contribute to susceptibility to BDM were not previously identified. A number of studies on other plant pathosystems have identified Arabidopsis *DMR6* and its orthologs in tomato and potato as S genes required for infection of multiple pathogens, including three plant pathogenic oomycetes [29, 32, 33]. In this study, we targeted the sweet basil homolog of *DMR6*, *ObDMR6*, for mutagenesis using CRISPR/Cas9. We were able to generate transgene-free knockout mutants of this multi-copy gene. Knocking out *ObDMR6* resulted in enhanced basil downy mildew resistance as evidenced by reduced accumulation of pathogen biomass and production of sporangia (Fig 6), suggesting the significant role *ObDMR6* plays in the compatibility of the non-model sweet basil-*P*. *belbahrii* pathosystem. These transgene-free gene edited lines with improved disease resistance have the potential to be used in sweet basil production upon further evaluation.

*ObDMR6* appears to have numerous copies in the genome of Genoveser (Fig 1). From our initial experiment to sequence and identify *ObDMR6* in this cultivar, we sequenced a total of 13 clones that contained *ObDMR6* gDNA and identified six distinct variants. When we employed the amplicon deep sequencing of the 417 bp *ObDMR6* fragment from transgene-free *Obdmr6* T1 lines S1:3–8, S1:49–9 and S1S2:15–6, we also sequenced this fragment from a WT plant.

A total of 451,059 reads were generated, and the unique sequences that accounted for over 1% of total reads were analyzed. 8 unique sequences were detected, with the sequence corresponding to *ObDMR6 v1* as the most abundant one (38.95% reads). The other seven unique sequences share SNPs ranging 1–8 bp. Considering this fragment is only a small portion of the *ObDMR6* gDNA and polymorphisms exist in sequences outside of this region (S2 Appendix), the number of *ObDMR6* unique sequences could be much higher. Even taking into account that sweet basil is allotetraploid [4], the high number of *ObDMR6* unique sequences with high homology suggests that there have been massive duplication events in the genome.

Despite the high copy number of *ObDMR6* in Genoveser, near-complete knockout was achieved in multiple T0 transgenic plants, setting another example of sweet basil gene editing with high mutation efficiency using the protocols previously established by Navet and Tian [24]. This study demonstrates the capacity of this highly efficient *Agrobacterium*-mediated CRISPR/Cas9 gene editing system to generate mutants for functional genomics studies of genes with high copy number or belonging to multi-gene families with redundant functions in tetraploid sweet basil without the need of gene editing in multiple successive generations.

For the T0 transgenic lines carrying two sgRNAs, only four carried mutations at S2 target site while 20 contained mutations at S1 target site. The four lines with mutations at S2 also contained mutations at S1 target, with much higher indel percentage at the latter. These results clearly showed that mutation mediated by sgRNA2 was much less efficient than sgRNA1. Similar results were observed when pKSE401 was used to express two sgRNAs for gene editing of *ObDMR1* [24]. In both cases, the sgRNA with low mutation efficiency was expressed under the control of U6-29p promoter, while the one with high mutation efficiency was driven by U6-26p. Other factors related to sgRNA target sequence and genomic context of the target site may have contributed to the low mutation efficiency. However, the fact that the sgRNAs driven by U6-29p mediated low mutation efficiency in both cases may suggest that this promoter is not suitable for use in sweet basil. Further experiments to determine this would benefit future construct design when multiplexing gene editing of sweet basil is required.

In order to detect mutations in *ObDMR6* rapidly and cost effectively, we employed Synthego’s ICE v2 program to decode the Sanger sequencing chromatograms of a *ObDMR6* fragment amplified from transgenic lines. The accuracy of the program was cross-checked with the high-throughput amplicon deep sequencing of the same target region for multiple transgene-free T1 lines. The results from both methods were overall consistent except that ICE v2 did not specify the inserted nucleotides in S1:3–8 and S1:49–9, failed to identify the indels of minor occurrence in S1:49–9, and decoded a small percentage of 3 bp deletion with deletion of different nucleotides in S1:3–8 (Fig 5). Despite the vast complexity contributed by the allotetraploidy of sweet basil and high copy number of *ObDMR6* in Genoveser genome, ICE v2 was able to determine the major mutation types and their abundance largely agreeable with the data generated through amplicon deep sequencing which represents an accurate mutation detection method. These results demonstrated the power of ICE v2’s algorithm in mutation detection of gene edited organisms.

We performed the pathogen infection assays of transgene-free *Obdmr6* mutants and WT plants with abundant fresh inocula under lab conditions that were very conducive to *P*. *belbahrii* infection and sporulation. In repeated assays, *Obdmr6* mutants consistently supported significantly lower amount of pathogen growth and sporulation than WT plants (Fig 6). However, *Obdmr6* mutants also got infected and produced a substantial amount of sporangia. It is not unusual that high disease pressure diminishes disease resistance. This is the case for the recently bred downy mildew resistant sweet basil varieties through traditional breeding [14]. It is very likely that field conditions generate less disease pressure than we put in our lab infection assays, considering the factors that negatively affect pathogen infection and disease development in the natural environments, such as less available source of fresh inocula, strong sunlight, wind blow, and the ever-changing and the highly variable conditions generated by macro and microclimates. The next step is to perform field trials to evaluate the resistance level of *Obdmr6* mutants and their potentials to be used in agricultural production. Given the significant reduction in pathogen biomass and sporangia produced under favorable conditions, it is likely that we will see a greater difference in disease resistance between the *Obdmr6* mutants and WT in field conditions that are less favorable for the pathogen. In that case, these transgene-free *Obdmr6* mutant lines will provide growers with additional choices of varieties to combat the devastating basil down mildew disease.

## Supporting information

S1 Appendix Transcriptomic sequences of sweet basil cultivar Dolly. (FASTA)

S2 Appendix Alignment of the gDNA sequences of six *ObDMR6* variants with a *ObDMR6* cDNA sequence showing the exons and introns, and the conservation among *ObDMR6* variants of sgRNA target sites and primers used for amplifying the *ObDMR6* fragments for mutation analyses. (DOCX)

S3 Appendix Alignment of the protein coding sequences of three transcripts that encode ObDMR6 from sweet basil cultivar Dolly. (DOCX)

S4 Appendix Predicted secondary structure of sgRNA target sequences. (DOCX)

S5 Appendix Total indel percentage, indel types and the corresponding percentage in T0 transgenic lines. (XLSX)

S1 Raw images (PDF)
