# Supplementary material for: CRISPR/Cas9-mediated mutagenesis of sweet basil candidate susceptibility gene ObDMR6 enhances downy mildew resistance
Source: PLoS One. 2021 Jun 10;16(6):e0253245. doi: 10.1371/journal.pone.0253245 (PMC8191900; doi:10.1371/journal.pone.0253245)
Supplement: S2 Appendix — (DOCX) [file pone.0253245.s002.docx]

**S2 Appendix. Alignment of the gDNA sequences of six *ObDMR6* variants with a *ObDMR6* cDNA sequence**. Exons are highlighted in green. The 20-nt target sequences of two sgRNAs, and primer sequences used for amplify the ObDMR6 fragments for mutation analyses are indicated.

v1_ObDMR6 ATGGAAACGAAGGTCATTAGTGGAACACAGTTCGCAAGCCTGCCGAGTTGCTATGTCCGTCCAGAATCCGAGAGGCCTAA

v2_ObDMR6 ATGGAAACGAAGGTCATTAGTGGAACACAGTTCGCAAGCCTGCCGAGTTGCTATGTCCGTCCAGAATCCGAGAGGCCTAA

v3_ObDMR6 ATGGAAACGAAGGTCATTAGTGGAACACAGTTCACAAGCCTCCCGAGTTGCTATGTCCGTCCAGAATCTGAGAGGCCAAA

v4_ObDMR6 ATGGAAACGAAGGTCATTAGTGGAACACAGTTCGCAAGCCTGCCGAGTTGCTATGTCCGTCCAGAATCCGAGAGGCCTAA

v5_ObDMR6 ATGGAAACGAAGGTCATTAGTGGAACACAGTTCACAAGCCTCCCGAGTTGCTATGTCCGTCCAGAATCTGAGAGGCCAAA

v6_ObDMR6 ATGGAAACGAAGGTCATTAGTGGAACACAGTTCACAAGCCTCCCGAGTTGCTATGTCCGTCCAGAATCTGAGAGGCCAAA

cDNA_ObDMR6 ATGGAAACGAAGGTCATTAGTGGAACACAGTTCACAAGCCTCCCGAGTTGCTATGTCCGTCCAGAATCTGAGAGGCCAAA

********************************* ******* ************************** ******** **

v1_ObDMR6 GTTATCTGAAGTTGCTGATTGCGAAGATGTTCCCGTCATTGATTTGGGCTGCGGAGATCGTAGCCTGATAGTCAAACAGA

v2_ObDMR6 GTTATCTGAAGTTGCTGATTGCGAAGATGTTCCCGTCATTGATTTGGGCTGCGGAGATCGTAGCCTGATAGTCAAACAGA

v3_ObDMR6 GTTATCTGAAGTTGCTGATTGCGAAGATGTTCCCGTCATTGATTTGGGCTGCGGAGATCGTGGCCTAATAGTCAAACAGA

v4_ObDMR6 GTTATCTGAAGTTGCTGATTGCGAAGATGTTCCCGTCATTGATTTGGGCTGCGGAGATCGTAGCCTGATAGTCAAACAGA

v5_ObDMR6 GTTATCTGAAGTTGCTGATTGCGAAGATGTTCCCGTCATTGATTTGGGCTGCGGAGATCGTGGCCTAATAGTCAAACAGA

v6_ObDMR6 GTTATCTGAAGTTGCTGATTGCGAAGATGTTCCCGTCATTGATTTGGGCTGCGGAGATCGTGGCCTAATAGTCAAACAGA

cDNA_ObDMR6 GTTATCTGAAGTTGCTGATTGCGAAGATGTTCCCGTCATTGATTTGGGCTGCGGAGATCGTGGCCTAATAGTCAAACAGA

************************************************************* **** *************

v1_ObDMR6 TCGGTGATGCTTGTCGAGAATATGGATTTTTCCAGGTTACTTAATGTTTG---AACGTACCTCAGCTACTCGTTGAACCA

v2_ObDMR6 TCGGTGATGCTTGTCGAGAATATGGATTTTTCCAGGTTACTTAATGTTTG---AACGTACCTCAGCTACTCGTTGAACCA

v3_ObDMR6 TCGGTGATGCTTGTCGAGAATATGGATTTTTCCAGGTTAGTTAGTTAATGTACAACGTAACTCAGCTATTCGCTGAACCA

v4_ObDMR6 TCGGTGATGCTTGTCGAGAATATGGATTTTTCCAGGTTACTTAATGTTTG---AACGTACCTCAGCTACTCGTTGAACCA

v5_ObDMR6 TCGGTGATGCTTGTCGAGAATATGGATTTTTCCAGGTTAGTTAGTTAATGTACAACGTAACTCAGCTATTCGCTGAACCA

v6_ObDMR6 TCGGTGATGCTTGTCGAGAATATGGATTTTTCCAGGTTAGTTAGTTAATGTACAACGTAACTCAGCTATTCGCTGAACCA

cDNA_ObDMR6 TCGGTGATGCTTGTCGAGAATATGGATTTTTCC-----------------------------------------------

*********************************

v1_ObDMR6 CTAGTCCA-AGTGCTGATTTCGTTTCCTCTTTTGGATGTATTCTGTCAGGTGATCAATCATGCCGTGCCGAAAGACATAG

v2_ObDMR6 CTAGTCCA-AGTGCTGATTTCGTTTCCTCTTTTGGATGTATTCTGTCAGGTGATCAATCATGCCGTGCCGAAAGACATAG

v3_ObDMR6 CTAGTCCAAAGTGCTAATTTCTTTTCCTCGTTTGAATGTATTCTATCAGGTGATCAATCATGCAGTGCCGAAAGACATAG

v4_ObDMR6 CTAGTCCA-AGTGCTGATTTCGTTTCCTCTTTTGGATGTATTCTGTCAGGTGATCAATCATGCCGTGCCGAAAGACATAG

v5_ObDMR6 CTAGTCCAAAGTGCTAATTTCTTTTCCTCGTTTGAATGTATTCTATCAGGTGATCAATCATGCAGTGCCGAAAGACATAG

v6_ObDMR6 CTAGTCCAAAGTGCTAATTTCTTTTCCTCGTTTGAATGTATTCTATCAGGTGATCAATCATGCAGTGCCGAAAGACATAG

cDNA_ObDMR6 -----------------------------------------------AGGTGATCAATCATGCAGTGCCGAAAGACATAG

**************** ****************

v1_ObDMR6 TGGATAAAATGGTGGGGGTGGCGCATGAATTCTTCAGTCTATCTGTGGAGGAGAAGATGAAATTATACTCTGATGACCCT

v2_ObDMR6 TGGATAAAATGGTGGGGGTGGCGCATGAATTCTTCAGTCTATCTGTGGAGGAGAAGATGAAATTATACTCTGATGACCCT

v3_ObDMR6 TGGATAAAATGGTGGCGGTGGCGCATGAATTCTTCAGTCTATCCGTGGAGGAGAAGATGAAATTATACTCTGATGACCCT

v4_ObDMR6 TGGATAAAATGGTGGGGGTGGCGCATGAATTCTTCAGTCTATCTGTGGAGGAGAAGATGAAATTATACTCTGATGACCCT

v5_ObDMR6 TGGATAAAATGGTGGCGGTGGCGCATGAATTCTTCAGTCTATCTGTGGAGGAGAAGATGAAATTATACTCTGATGACCCT

v6_ObDMR6 TGGATAAAATGGTGGCGGTGGCGCATGAATTCTTCAGTCTATCCGTGGAGGAGAAGATGAAATTATACTCTGATGACCCT

cDNA_ObDMR6 TGGATAAAATGGTGGCGGTGGCGCATGAATTCTTCAGTCTATCCGTGGAGGAGAAGATGAAATTATACTCTGATGACCCT

*************** *************************** ************************************

ObDMR6-S1S2_F1 ObDMR6-S1S2_F2

v1_ObDMR6 TCAAAAACGATGCGACTCTCTACGAGTTTCAACGTTAGAAAGGAGACCGTTCACAACTGGAGAGACTATCTCAGGCTTCA

v2_ObDMR6 TCAAAAACGATGCGACTCTCTACGAGTTTCAACGTTAGAAAGGAGACCGTTCACAACTGGAGAGACTATCTCAGGCTTCA

v3_ObDMR6 TCCAAAACTATGCGACTCTCCACGAGTTTCAACGTTAGAAAGGAGACCGTACACAACTGGAGAGACTATCTCAGGCTTCA

v4_ObDMR6 TCAAAAACGATGCGACTCTCTACGAGTTTCAACGTTAGAAAGGAGACCGTTCACAACTGGAGAGACTATCTCAGGCTTCA

v5_ObDMR6 TCAAAAACGATGCGACTCTCTACGAGTTTCAACGTTAGAAAGGAGACCGTTCACAACTGGAGAGACTATCTCAGGCTTCA

v6_ObDMR6 TCCAAAACTATGCGACTCTCCACGAGTTTCAACGTTAGAAAGGAGACCGTACACAACTGGAGAGACTATCTCAGGCTTCA

cDNA_ObDMR6 TCCAAAACTATGCGACTCTCCACGAGTTTCAACGTTAGAAAGGAGACCGTACACAACTGGAGAGACTATCTCAGGCTTCA

** ***** *********** ***************************** *****************************

v1_ObDMR6 TTGCTACCCCTTGGAGAAATACGCGCCTGAATGGCCGTCTAATCCCTCTTCTTTCAAGTAAGCCAACCTGTTTTCTTAGT

v2_ObDMR6 TTGCTACCCCTTGGAGAAATACGCGCCTGAATGGCCGTCTAATCCCTCTTCTTTCAAGTAAGCCAACCTGTTTTCTTAGT

v3_ObDMR6 TTGTTACCCCTTGGAGAAATACGCGCCTGAATGGCCATCTAATCCCTCTTCTTTCAAGTAAGCCAACCTGTTTTCTTAGT

v4_ObDMR6 TTGCTACCCCTTGGAGAAATACGCGCCTGAATGGCCGTCTAATCCCTCTTCTTTCAAGTAAGCCAACCTGTTTTCTTAGT

v5_ObDMR6 TTGCTACCCCTTGGAGAAATACGCGCCTGAATGGCCGTCTAATCCCTCTTCTTTCAAGTAAGCCAACCTGTTTTCTTAGT

v6_ObDMR6 TTGTTACCCCTTGGAGAAATACGCGCCTGAATGGCCATCTAATCCCTCTTCTTTCAAGTAAGCCAACCTGTTTTCTTAGT

cDNA_ObDMR6 TTGTTACCCCTTGGAGAAATACGCGCCTGAATGGCCATCTAATCCCTCTTCTTTCAA-----------------------

*** ******************************** ********************

sgRNA1

v1_ObDMR6 AGTGCCAGCAAAAGATTGTTGATATGAATCGCATTTTCATTTGTAGGGATATCGTAAGCACATACTGCAAAGAAGTT**CGG**

v2_ObDMR6 AGTGCCAGCAAAAGATTGTTGATATGAATCGCATTTTCATTTGTAGGGATATCGTAAGCACATACTGCAAAGAAGTT**CGG**

v3_ObDMR6 AGTGCCAGCAAAAGATTTTTGAGATGAATCGTATTTTCATTTGTAGGGATATCGTAAGCACATACTGCAAAGAAGTT**CGG**

v4_ObDMR6 AGTGCCAGCAAAAGATTGTTGATATGAATCGCATTTTCATTTGTAGGGATATCGTAAGCACATACTGCAAAGAAGTT**CGG**

v5_ObDMR6 AGTGCCAGCAAAAGATTGTTGATATGAATCGCATTTTCATTTGTAGGGATATCGTAAGCACATACTGCAAAGAAGTT**CGG**

v6_ObDMR6 AGTGCCAGCAAAAGATTTTTGAGATGAATCGTATTTTCATTTGTAGGGATATCGTAAGCACATACTGCAAAGAAGTT**CGG**

cDNA_ObDMR6 ----------------------------------------------GGATATCGTAAGCACATACTGCAAAGAAGTT**CGG**

**********************************

v1_ObDMR6 GCCCTGGGATTCTGGTTGCAAGAGGCCATATCGGAGAGCCTCGGTTTACACAAAGACTGCCTCAAGAATGTATTGGGAGA

v2_ObDMR6 GCCCTGGGATTCTGGTTGCAAGAGGCCATATCGGAGAGCCTCGGTTTACACAAAGACTGCCTCAAGAATGTATTGGGAGA

v3_ObDMR6 GCCCTGGGATTCTGGTTGCAAGAGGCCATATCGGAGAGCCTCGGTTTACACAAAGACTGCCTCAAGAATGTATTGGGAGA

v4_ObDMR6 GCCCTGGGATTCTGGTTGCAAGAGGCCATATCGGAGAGCCTCGGTTTACACAAAGACTGCCTCAAGAATGTATTGGGAGA

v5_ObDMR6 GCCCTGGGATTCTGGTTGCAAGAGGCCATATCGGAGAGCCTCGGTTTACACAAAGACTGCCTCAAGAATGTATTGGGAGA

v6_ObDMR6 GCCCTGGGATTCTGGTTGCAAGAGGCCATATCGGAGAGCCTCGGTTTACACAAAGACTGCCTCAAGAATGTATTGGGAGA

cDNA_ObDMR6 GCCCTGGGATTCTGGTTGCAAGAGGCCATATCGGAGAGCCTCGGTTTACACAAAGACTGCCTCAAGAATGTATTGGGAGA

********************************************************************************

sgRNA2

3’-**GGT**CTAGACTGAAAGCCTAATGG-5’

v1_ObDMR6 GCAAGGGCAACACATGGCCATCAACTTCTATCCTGCATGCCCAGAA**CCA**GATCTGACTTTCGGATTACCCGCTCATACAG

v2_ObDMR6 GCAAGGGCAACACATGGCCATCAACTTCTATCCTGCATGCCCAGAA**CCA**GATCTGACTTTCGGATTACCCGCTCATACAG

v3_ObDMR6 GCAAGGGCAACATATGGCCATCAACTTTTATCCTGCATGCCCAGAA**CCA**GATCTGACTTTCGGATTACCCGCTCATACAG

v4_ObDMR6 GCAAGGGCAACATATGGCCATCAACTTTTATCCTGCATGCCCAGAA**CCA**GATCTGACTTTCGGATTACCCGCTCATACAG

v5_ObDMR6 GCAAGGGCAACACATGGCCATCAACTTCTATCCTGCATGCCCAGAA**CCA**GATCTGACTTTCGGATTACCCGCTCATACAG

v6_ObDMR6 GCAAGGGCAACATATGGCCATCAACTTTTATCCTGCATGCCCAGAA**CCA**GATCTGACTTTCGGATTACCCGCTCATACAG

cDNA_ObDMR6 GCAAGGGCAACATATGGCCATCAACTTTTATCCTGCATGCCCAGAA**CCA**GATCTGACTTTCGGATTACCCGCTCATACAG

************ ************** ****************************************************

ObDMR6-S1S2_R2

3’-GAAGTCCAAGAGTTCCTACCCTT-5’

v1_ObDMR6 ATCCGAATGCGCTCACCATTCTCCTTCAAGATTTACTGGTTTCGGGTCTTCAGGTTCTCAAGGATGGGAAATGGTTAGCA

v2_ObDMR6 ATCCGAATGCGCTCACCATTCTCCTTCAAGATTTACTGGTTTCGGGTCTTCAGGTTCTCAAGGATGGGAAATGGTTAGCA

v3_ObDMR6 ATCCGAATGCACTCACCATTCTCCTTCAAGATTTACTGGTTTCGGGTCTTCAGGTTCTCAAGGATGGGAAATGGTTAGCA

v4_ObDMR6 ATCCGAATGCACTCACCATTCTCCTTCAAGATTTACTGGTTTCGGGTCTTCAGGTTCTCAAGGATGGGAAATGGTTAGCA

v5_ObDMR6 ATCCGAATGCACTCACCATTCTCCTTCAAGATTTACTGGTTTCGGGTCTTCAGGTTCTCAAGGATGGGAAATGGTTAGCA

v6_ObDMR6 ATCCGAATGCACTCACCATTCTCCTTCAAGATTTACTGGTTTCGGGTCTTCAGGTTCTCAAGGATGGGAAATGGTTAGCA

cDNA_ObDMR6 ATCCGAATGCACTCACCATTCTCCTTCAAGATTTACTGGTTTCGGGTCTTCAGGTTCTCAAGGATGGGAAATGGTTAGCA

********** *********************************************************************

ObDMR6-S1S2_R1

3’-GGTCTACGAAAACAGTAGTTGTAAC-5’

v1_ObDMR6 ATAAAGCCCCAGCCAGATGCTTTTGTCATCAACATTGGTGATCAAATCCAGGTGAACACTATGTGTACAATTGTTATGGA

v2_ObDMR6 ATAAAGCCCCAGCCAGATGCTTTTGTCATCAACATTGGTGATCAAATCCAGGTGAACACTATTTGTACAATTGTTA----

v3_ObDMR6 ATAAAGCCCCAGCCAGATGCTTTTGTCATCAACATTGGTGATCAAATCCAGGTGACCACTATTTGTACAATTGTTA----

v4_ObDMR6 ATAAAGCCCCAGCCAGATGCTTTTGTCATCAACATTGGTGATCAAATCCAGGTGACCACTATTTGTACAATTGTTA----

v5_ObDMR6 ATAAAGCCCCAGCCAGATGCTTTTGTCATCAACATTGGTGATCAAATCCAGGTGAACACTATTTGTACAATTGTTA----

v6_ObDMR6 ATAAAGCCCCAGCCAGATGCTTTTGTCATCAACATTGGTGATCAAATCCAGGTGACCACTATTTGTACAATTGTTA----

cDNA_ObDMR6 ATAAAGCCCCAGCCAGATGCTTTTGTCATCAACATTGGTGATCAAATC--------------------------------

************************************************

v1_ObDMR6 AATTCATTCTACTGATTATGTAAGAATGCCCT-TATGGAAAATGCATTTGTGTCCCTTTGCCCACAAAAACTGCGATTCG

v2_ObDMR6 ------------------TGTAAGAATGCCCT-TATGGA----------------------------AAACTGCGATTCG

v3_ObDMR6 ------------------TGTAAGAATGCCCTTTATGGA----------------------------AAACTGCGATTCG

v4_ObDMR6 ------------------TGTAAGAATGCCCTTTATGGA----------------------------AAACTGCGATTCG

v5_ObDMR6 ------------------TGTAAGAATGCCCT-TATGGA----------------------------AAACTGCGATTCG

v6_ObDMR6 ------------------TGTAAGAATGCCCTTTATGGA----------------------------AAACTGCGATTCG

cDNA_ObDMR6 --------------------------------------------------------------------------------

v1_ObDMR6 GGTAAGATTTAGGGGAAAGAGGATGAATCATCATCTTACTGTTTCACGAATTAGGGGATTTTATCCCACCATTTGAAGTT

v2_ObDMR6 GGTAAGATCTAGGGGAAAGAGGATGAATCCCTGTCTTACAGTTTCACGAATTAGGGGATTTTATCCCACAAT--------

v3_ObDMR6 GGTAAGATCTAGGGGAAAGAGGATGAATCCTTGTCTTACTGTTTCACGAATTAGGGGATTTTATCCCACAAT--------

v4_ObDMR6 GGTAAGATCTAGGGGAAAGAGGATGAATCCTTGTCTTACTGTTTCACGAATTAGGGGATTTTATCCCACAAT--------

v5_ObDMR6 GGTAAGATCTAGGGGAAAGAGGATGAATCCCTGTCTTACAGTTTCACGAATTAGGGGATTTTATCCCACAAT--------

v6_ObDMR6 GGTAAGATCTAGGGGAAAGAGGATGAATCCTTGTCTTACTGTTTCACGAATTAGGGGATTTTATCCCACAAT--------

cDNA_ObDMR6 --------------------------------------------------------------------------------

v1_ObDMR6 GGGGTAAATTATCCTATAGTTTATTTCCATTAGAGTATTTTACCCTCCGTGAATAACGATGTCAACTGTTTTGTCACGTC

v2_ObDMR6 --------------------------------------------------------------------------------

v3_ObDMR6 --------------------------------------------------------------------------------

v4_ObDMR6 --------------------------------------------------------------------------------

v5_ObDMR6 --------------------------------------------------------------------------------

v6_ObDMR6 --------------------------------------------------------------------------------

cDNA_ObDMR6 --------------------------------------------------------------------------------

v1_ObDMR6 ACACCTTTAATTCCCGCGTGGAAAAAAATAATTCTTTTTTCCAGGGTATTGGACGAAAATAAAATCGTTTTGTTTAACGT

v2_ObDMR6 --------------------------------------------------------------------------------

v3_ObDMR6 --------------------------------------------------------------------------------

v4_ObDMR6 --------------------------------------------------------------------------------

v5_ObDMR6 --------------------------------------------------------------------------------

v6_ObDMR6 --------------------------------------------------------------------------------

cDNA_ObDMR6 --------------------------------------------------------------------------------

v1_ObDMR6 CGTCCCTAATATCGATTTATGGGAAATTCAGGAAAATTATCCGTTCTCACAAGTCATGTTCCAACGTTTCCATTAATATG

v2_ObDMR6 --------------------------------------------------------------------------------

v3_ObDMR6 --------------------------------------------------------------------------------

v4_ObDMR6 --------------------------------------------------------------------------------

v5_ObDMR6 --------------------------------------------------------------------------------

v6_ObDMR6 --------------------------------------------------------------------------------

cDNA_ObDMR6 --------------------------------------------------------------------------------

v1_ObDMR6 GGTTGAACTGCGTCGTTTTCGTTCAATGCCCTTAAAATAAAGAACTTAATATTTTTTCCACGCTGGCATTTAACGTGTGA

v2_ObDMR6 -----------------TTCGTTCAATGCCCTAAAAATAAAGA--T-TAATATTTTTCCACGCTGGAATTTAATGTGTGA

v3_ObDMR6 -----------------TTCGTTCAATGCCCTAAAAATAAAGAAAT-TAATATTTTTCCACGCTGGAATTTAATGTGTGA

v4_ObDMR6 -----------------TTCGTTCAATGCCCTAAAAATAAAGAAAT-TAATATTTTTCCACGCTGGAATTTAATGTGTGA

v5_ObDMR6 -----------------TTCGTTCAATGCCCTAAAAATAAAGA--T-TAATATTTTTCCACGCTGGAATTTAATGTGTGA

v6_ObDMR6 -----------------TTCGTTCAATGCCCTAAAAATAAAGAAAT-TAATATTTTTCCACGCTGGAATTTAATGTGTGA

cDNA_ObDMR6 --------------------------------------------------------------------------------

v1_ObDMR6 AGTAACGAATGTTTGACATAGTCATACATGGAGGATAGAATACCCTAAAAATCCCCTGAGAAGTGAAACAGTAAGATAAC

v2_ObDMR6 AGTAAAAAATATTTGACATCGTCATTCATGG------------------------------AGTGAAACAGTAAGATAAC

v3_ObDMR6 AGTAAAAAATATTTGACATCGTCATTCATGG------------------------------GGTGAAACAGTAAGATAAC

v4_ObDMR6 AGTAAAAAATATTTGACATCGTCATTCATGG------------------------------GGTGAAACAGTAAGATAAC

v5_ObDMR6 AGTAAAAAATATTTGACATCGTCATTCATGG------------------------------AGTGAAACAGTAAGATAAC

v6_ObDMR6 AGTAAAAAATATTTGACATCGTCATTCATGG------------------------------AGTGAAACAGTAAGATAAC

cDNA_ObDMR6 --------------------------------------------------------------------------------

v1_ObDMR6 TTACCCTTCACGATTCGTCGTGTGTTTTCAGATCGGAGGAGCATTGCTCTTTTTATCTACTGAACAAACTTATCAGTTCA

v2_ObDMR6 TTACCCTTCATGATTCGTCGTGTTTTTTCAGATCGGAGGAGCATGGCTCTTTTTATCTACTGAACAAACTTATCAGTTCA

v3_ObDMR6 TTACCCTTCATGATTCGTCGTGTTTTTTCAGATCGGAGGAGCATGGCTCTTTTTATCTACTGAACAAACTTATCAGTTCA

v4_ObDMR6 TTACCCTTCATGATTCGTCGTGTTTTTTCAGATCGGAGGAGCATGGCTCTTTTTATCTACTGAACAAACTTATCAGTTCA

v5_ObDMR6 TTACCCTTCATGATTCGTCGTGTTTTTTCAGATCGGAGGAGCATGGCTCTTTTTATCTACTGAACAAACTTATCAGTTCA

v6_ObDMR6 TTACCCTTCATGATTCGTCGTGTTTTTTCAGATCGGAGGAGCATGGCTCTTTTTATCTACTGAACAAACTTATCAGTTCA

cDNA_ObDMR6 --------------------------------------------------------------------------------

v1_ObDMR6 GATGCATAAGAAAAACAGTTTTAGCATCTCCTTACTGAACTATCTGTGCAACTCACTTCCGCAGGCATTCAGTAATGGGA

v2_ObDMR6 GATGCATAAGAAAAACAGTTTTAGCATCTCCTTACTGAACTATCTGTGCAACTCACTTCCGCAGGCATTCAGTAATGGGA

v3_ObDMR6 GATGCATAAGAAAAACTATTTTAGCATCTCCTTACTGAACTCTCTCTGCAACTCACTTCTGCAGGCATTCAGTAATGGGA

v4_ObDMR6 GATGCATAAGAAAAACTATTTTAGCATCTCCTTACTGAACTCTCTCTGCAACTCACTTCTGCAGGCATTCAGTAATGGGA

v5_ObDMR6 GATGCATAAGAAAAACAGTTTTAGCATCTCCTTACTGAACTATCTGTGCAACTCACTTCTGCAGGCATTTAGTAATGGGA

v6_ObDMR6 GATGCATAAGAAAAACAGTTTTAGCATCTCCTTACTGAACTATCTGTGCAACTCACTTCTGCAGGCATTTAGTAATGGGA

cDNA_ObDMR6 -------------------------------------------------------------CAGGCATTCAGTAATGGGA

******** **********

v1_ObDMR6 AGTACAGAAGCGTGTGGCATCGAGCTGTCGTAAATTCGAACAAAGCCAGACTCTCGGTCGCTTCATTCCTCTGCCCGTGC

v2_ObDMR6 AGTACAGAAGCGTGTGGCATCGAGCTGTCGTAAATTCGAACAAAGCCAGACTCTCGGTCGCTTCATTCCTCTGCCCGTGC

v3_ObDMR6 AGTACAGAAGCGTGTGGCATCGAGCCGTCGTAAATTCAAACAAAGCTAGACTCTCGGTTGCTTCGTTCCTCTGCCCGTGT

v4_ObDMR6 AGTACAGAAGCGTGTGGCATCGAGCCGTCGTAAATTCAAACAAAGCTAGACTCTCGGTTGCTTCGTTCCTCTGCCCGTGT

v5_ObDMR6 AGTACAGGAGCGTGTGGCATCGAGCCGTCGTAAATTCAAACAAAGCCAGACTCTCGGTCGCTTCGTTCCTCTGCCCGTGC

v6_ObDMR6 AGTACAGGAGCGTGTGGCATCGAGCCGTCGTAAATTCAAACAAAGCCAGACTCTCGGTCGCTTCGTTCCTCTGCCCGTGC

cDNA_ObDMR6 AGTACAGAAGCGTGTGGCATCGAGCCGTCGTAAATTCAAACAAAGCTAGACTCTCGGTTGCTTCGTTCCTCTGCCCGTGT

******* ***************** *********** ******** *********** ***** **************

v1_ObDMR6 GATGCAGCAAATATCAGCGCTCCAAATGAACTTACAACCGGCGATGATCGAGCAATATACAGAGGTTTTACATATGCCGA

v2_ObDMR6 GATGCAGCAAATATCAGCGCTCCAAATGAACTTACAACCGGCGATGATCGAGCAATATACAGAGGTTTTACATATGCCGA

v3_ObDMR6 GATGCAGCAAATATCAGCGCTCCAAATGAACTTACAACCGGCGATGATCGAGCAATATACAGAGGTTTTACATATGCCGA

v4_ObDMR6 GATGCAGCAAATATCAGCGCTCCAAATGAACTTACAACCGGCGATGATCGAGCAATATACAGAGGTTTTACATATGCCGA

v5_ObDMR6 GATGCAGCAAATATCAGCGCTCCAAATGAACTTACAACCGGCAATGATCGAGCAATATACAGAGGTTTTACATATGCCGA

v6_ObDMR6 GATGCAGCAAATATCAGCGCTCCAAATGAACTTACAACCGGCAATGATCGAGCAATATACAGAGGTTTTACATATGCCGA

cDNA_ObDMR6 GATGCAGCAAATATCAGCGCTCCAAATGAACTTACAACCGGCGATGATCGAGCAATATACAGAGGTTTTACATATGCCGA

****************************************** *************************************

v1_ObDMR6 GTACTACAAAAAGTTCTGGAGCCGGAACCTGGATCAGGAGCACTGCCTGGAACTATTCAAGAATTAG

v2_ObDMR6 GTACTACAAAAAGTTCTGGAGCCGGAACCTGGATCAGGAGCACTGCCTGGAACTATTCAAGAATTAG

v3_ObDMR6 GTACTACAAAAAGTTCTGGAGCCGGAACCTGGACCAGGAGCACTGCCTGGAACTATTCAAGAATTAG

v4_ObDMR6 GTACTACAAAAAGTTCTGGAGCCGGAACCTGGACCAGGAGCACTGCCTGGAACTATTCAAGAATTAG

v5_ObDMR6 GTACTACAAAAAGTTCTGGAGCCGGAACCTGGATCAGGAGCACTGCCTGGAACTATTCAAGAATTAG

v6_ObDMR6 GTACTACAAAAAGTTCTGGAGCCGGAACCTGGATCAGGAGCACTGCCTGGAACTATTCAAGAATTAG

cDNA_ObDMR6 GTACTACAAAAAGTTCTGGAGCCGGAACCTGGACCAGGAGCACTGCCTGGAACTATTCAAGAATTAG

********************************* *********************************
