# Supplementary material for: CRISPR/Cas9-mediated mutagenesis of sweet basil candidate susceptibility gene ObDMR6 enhances downy mildew resistance
Source: PLoS One. 2021 Jun 10;16(6):e0253245. doi: 10.1371/journal.pone.0253245 (PMC8191900; doi:10.1371/journal.pone.0253245)
Supplement: S3 Appendix — (DOCX) [file pone.0253245.s003.docx]

S3 Appendix. Alignment of the protein-encoding sequences of three transcripts that encode ObDMR6 from sweet basil cultivar Dolly.

comp38697_c1_seq2 ATGGAAACGAAGGTCATTAGTGGAACACAGTTCGCAAGCCTGCCGAGTTGCTATGTCCGT

comp38697_c1_seq3 ATGGAAACGAAGGTCATTAGTGGAACACAGTTCACAAGCCTCCCGAGTTGCTATGTCCGT

comp38697_c0_seq1 ATGGAAACGAAGGTCATTAGTGGAACACAGTTCGCAAGCCTGCCGAGTTGCTATGTCCGT

********************************* ******* ******************

comp38697_c1_seq2 CCAGAATCTGAGAGGCCAAAGTTATCTGAAGTTGCTGATTGCGAAGATGTTCCCGTCATT

comp38697_c1_seq3 CCAGAATCTGAGAGGCCAAAGTTATCTGAAGTTGCTGATTGCGAAGATGTTCCCGTCATT

comp38697_c0_seq1 CCAGAATCCGAGAGGCCTAAGTTATCTGAAGTTGCTGATTGCGAAGATGTTCCCGTCATT

******** ******** ******************************************

comp38697_c1_seq2 GATTTGGGCTGCGGAGATCGTGGCCTAATAGTCAAACAGATCGGTGATGCTTGTCGAGAA

comp38697_c1_seq3 GATTTGGGCTGCGGAGATCGTGGCCTAATAGTCAAACAGATCGGTGATGCTTGTCGAGAA

comp38697_c0_seq1 GATTTGGGCTGCGGAGATCGTAGCCTGATAGTCAAACAGATCGGTGATGCTTGTCGAGAA

********************* **** *********************************

comp38697_c1_seq2 TATGGATTTTTCCAGGTGATCAATCATGCAGTGCCGAAAGACATAGTGGATAAAATGGTG

comp38697_c1_seq3 TATGGATTTTTCCAGGTGATCAATCATGCAGTGCCGAAAGACATAGTGGATAAAATGGTG

comp38697_c0_seq1 TATGGATTTTTCCAGGTGATCAATCATGCAGTGCCGAAAGACATAGTGGATAAAATGGTG

************************************************************

comp38697_c1_seq2 GGGGTGGCGCATGAATTCTTCAGTCTATCTGTGGAGGAGAAGATGAAATTATACTCTGAT

comp38697_c1_seq3 GGGGTGGCGCATGAATTCTTCAGTCTATCTGTGGAGGAGAAGATGAAATTATACTCTGAT

comp38697_c0_seq1 GGGGTGGCGCATGAATTCTTCAGTCTATCCGTGGAGGAGAAGATGAAATTATACTCTGAT

***************************** ******************************

comp38697_c1_seq2 GACCCTTCCAAAACTATGCGACTCTCCACGAGTTTCAACGTTAGAAAGGAGACCGTACAC

comp38697_c1_seq3 GACCCTTCCAAAACTATGCGACTCTCCACGAGTTTCAACGTTAGAAAGGAGACCGTACAC

comp38697_c0_seq1 GACCCTTCCAAAACTATGCGACTCTCCACGAGTTTCAACGTTAGAAAGGAGACCGTTCAC

******************************************************** ***

comp38697_c1_seq2 AACTGGAGAGACTATCTCAGGCTTCATTGTTACCCCTTGGAGAAATACGCGCCTGAATGG

comp38697_c1_seq3 AACTGGAGAGACTATCTCAGGCTTCATTGTTACCCCTTGGAGAAATACGCGCCTGAATGG

comp38697_c0_seq1 AACTGGAGAGACTATCTCAGGCTTCATTGCTACCCCTTGGAGAAATACGCGCCTGAATGG

***************************** ******************************

comp38697_c1_seq2 CCATCTAATCCCTCTTCTTTCAAGGATATCGTAAGCACATACTGCAAAGAAGTTCGGGCC

comp38697_c1_seq3 CCATCTAATCCCTCTTCTTTCAAGGATATCGTAAGCACATACTGCAAAGAAGTTCGGGCC

comp38697_c0_seq1 CCATCTAATCCCTCTTCTTTCAAGGATATCGTAAGCACATACTGCAAAGAAGTTCGGGCC

************************************************************

comp38697_c1_seq2 CTGGGATTCTGGTTGCAAGAGGCCATATCGGAGAGCCTCGGTTTACACAAAGACTGCCTC

comp38697_c1_seq3 CTGGGATTCTGGTTGCAAGAGGCCATATCGGAGAGCCTCGGTTTACACAAAGACTGCCTC

comp38697_c0_seq1 CTGGGATTCTGGTTGCAAGAGGCCATATCGGAGAGCCTCGGTTTACACAAAGACTGCCTC

************************************************************

comp38697_c1_seq2 AAGAATGTATTGGGAGAGCAAGGGCAACATATGGCCATCAACTTTTATCCTGCATGCCCA

comp38697_c1_seq3 AAGAATGTATTGGGAGAGCAAGGGCAACATATGGCCATCAACTTTTATCCTGCATGCCCA

comp38697_c0_seq1 AAGAATGTATTGGGAGAGCAAGGGCAACACATGGCCATCAACTTCTATCCTGCATGCCCA

***************************** ************** ***************

comp38697_c1_seq2 GAACCAGATCTGACTTTCGGATTACCCGCTCATACAGATCCGAATGCGCTCACCATTCTC

comp38697_c1_seq3 GAACCAGATCTGACTTTCGGATTACCCGCTCATACAGATCCGAATGCGCTCACCATTCTC

comp38697_c0_seq1 GAACCAGATCTGACTTTCGGATTACCCGCTCATACAGATCCGAATGCACTCACCATTCTC

*********************************************** ************

comp38697_c1_seq2 CTTCAAGATTTACTGGTTTCGGGTCTTCAGGTTCTCAAGGATGGGAAATGGTTAGCAATA

comp38697_c1_seq3 CTTCAAGATTTACTGGTTTCGGGTCTTCAGGTTCTCAAGGATGGGAAATGGTTAGCAATA

comp38697_c0_seq1 CTTCAAGATTTACTGGTTTCGGGTCTTCAGGTTCTCAAGGATGGGAAATGGTTAGCAATA

************************************************************

comp38697_c1_seq2 AAGCCCCAGCCAGATGCTTTTGTCATCAACATTGGTGATCAAATCCAGGCATTCAGTAAT

comp38697_c1_seq3 AAGCCCCAGCCAGATGCTTTTGTCATCAACATTGGTGATCAAATCCAGGCATTCAGTAAT

comp38697_c0_seq1 AAGCCCCAGCCAGATGCTTTTGTCATCAACATTGGTGATCAAATCCAGGCATTCAGTAAT

************************************************************

comp38697_c1_seq2 GGGAAGTACAGAAGCGTGTGGCATCGAGC-------------------------------

comp38697_c1_seq3 GGGAAGTACAGAAGCGTGTGGCATCGAGC-------------------------------

comp38697_c0_seq1 GGGAAGTACAGAAGCGTGTGGCATCGAGCTGTCGTAAATTCGAACAAAGCCAGACTCTCG

*****************************

comp38697_c1_seq2 ------------------------------------------------------------

comp38697_c1_seq3 ------------------------------------------------------------

comp38697_c0_seq1 GTCGCTTCATTCCTCTGCCCGTGCGATGCAGCAAATATCAGCGCTCCAAATGAACTTACA

comp38697_c1_seq2 ------------------------------------------------------------

comp38697_c1_seq3 ------------------------------------------------------------

comp38697_c0_seq1 ACCGGCGATGATCGAGCAATATACAGAGGTTTTACATATGCCGAGTACTACAAAAAGTTC

comp38697_c1_seq2 ---------------------------------------------------

comp38697_c1_seq3 ---------------------------------------------------

comp38697_c0_seq1 TGGAGCCGGAACCTGGATCAGGAGCACTGCCTGGAACTATTCAAGAATTAG
