# Supplementary material for: CRISPR/Cas9-mediated mutagenesis of sweet basil candidate susceptibility gene ObDMR6 enhances downy mildew resistance
Source: PLoS One. 2021 Jun 10;16(6):e0253245. doi: 10.1371/journal.pone.0253245 (PMC8191900; doi:10.1371/journal.pone.0253245)

S4 Appendix. Predicted secondary structure of sgRNA target sequences using the web-based tool RNAStructure (<http://rna.urmc.rochester.edu/RNAstructureWeb/Servers/Predict1/Predict1.html>)


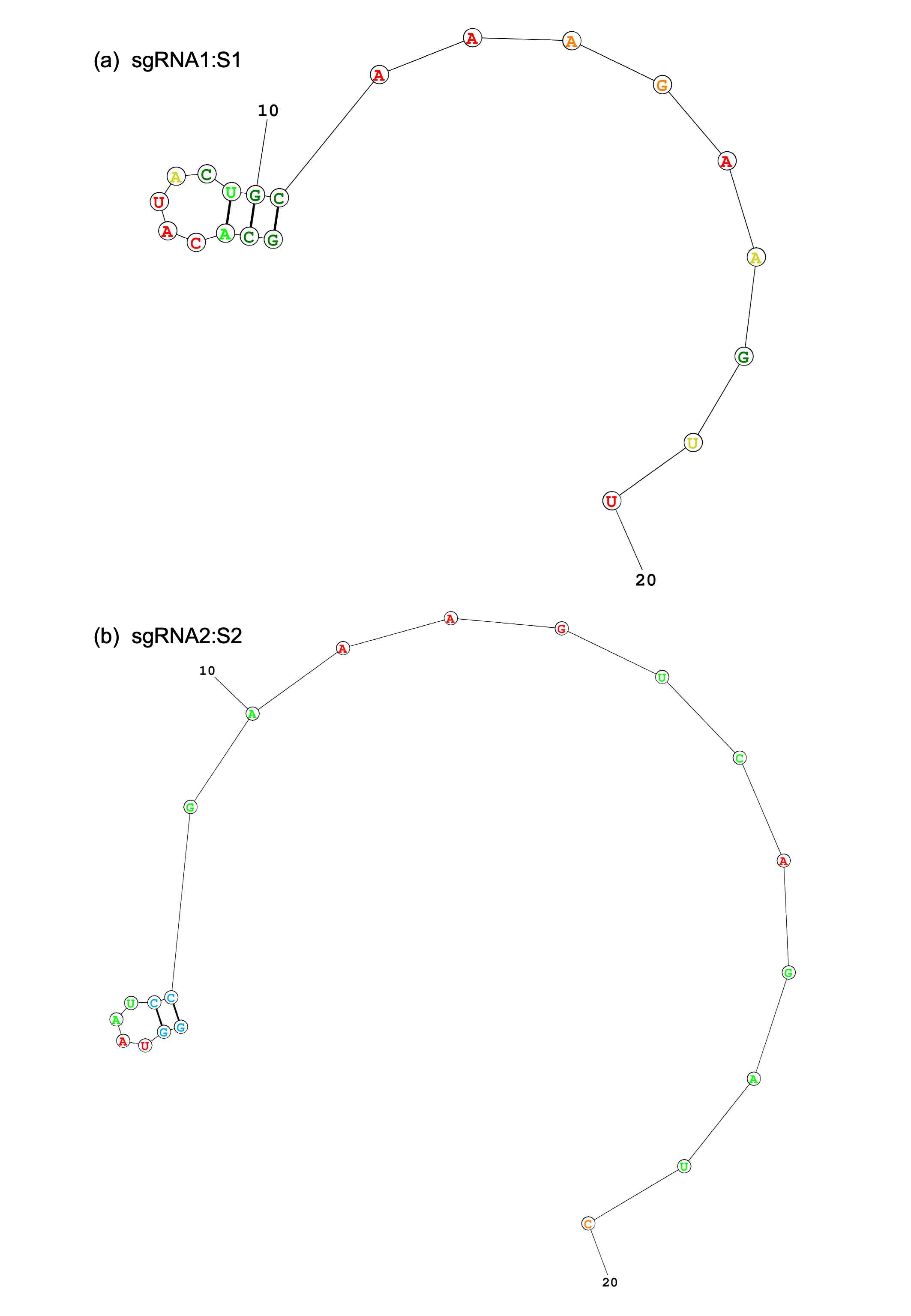

Supplement: S4 Appendix — (DOCX) [file pone.0253245.s004.docx]
