# Supplementary material for: CRISPR/Cas9-mediated mutagenesis of sweet basil candidate susceptibility gene ObDMR6 enhances downy mildew resistance
Source: PLoS One. 2021 Jun 10;16(6):e0253245. doi: 10.1371/journal.pone.0253245 (PMC8191900; doi:10.1371/journal.pone.0253245)

S1_raw_images for Figure 4. The labeling is the same as Figure 4 and the details are described in the legend of Figure 4.

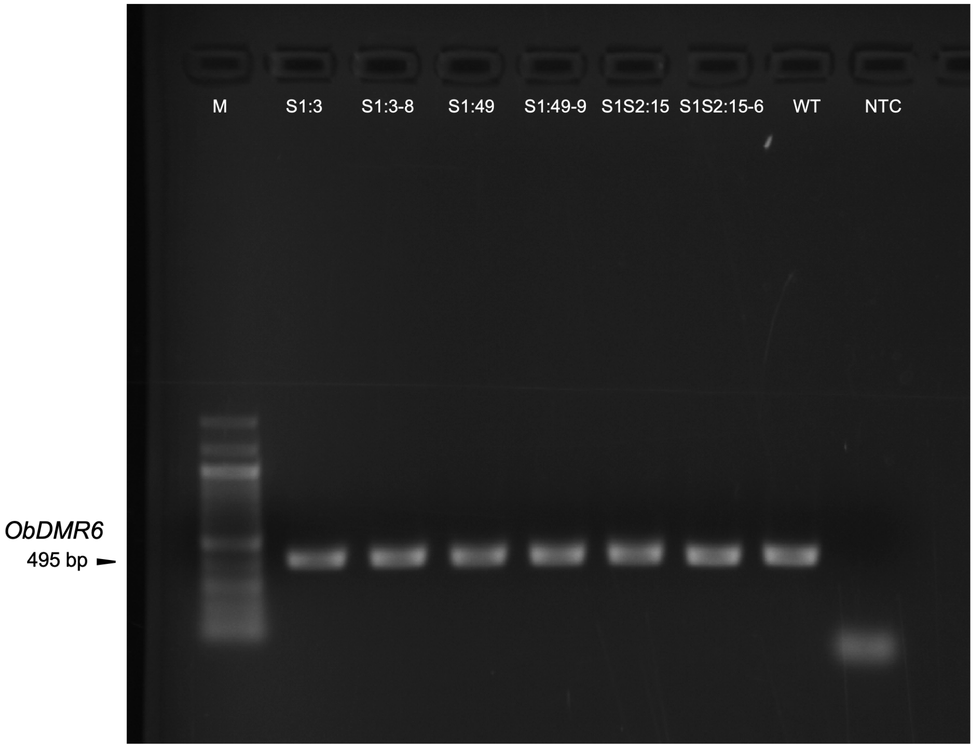

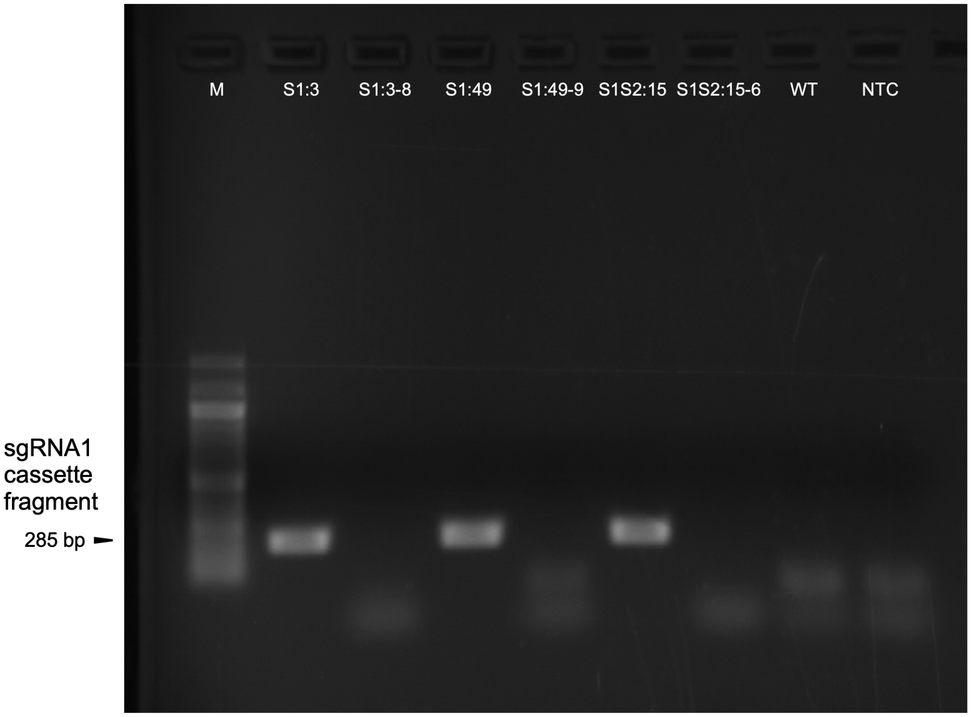

Supplement: S1 Raw images — (PDF) [file pone.0253245.s006.pdf]
